# Combination Chemotherapy with Selected Polyphenols in Preclinical and Clinical Studies—An Update Overview

**DOI:** 10.3390/molecules28093746

**Published:** 2023-04-26

**Authors:** Cvijeta Jakobušić Brala, Ana Karković Marković, Azra Kugić, Jelena Torić, Monika Barbarić

**Affiliations:** Faculty of Pharmacy and Biochemistry, University of Zagreb, A. Kovačića 1, 10 000 Zagreb, Croatia; cjakobus@pharma.unizg.hr (C.J.B.); akarkovic@pharma.unizg.hr (A.K.M.); akugic@student.pharma.hr (A.K.); jelenatoric@gmail.com (J.T.)

**Keywords:** cancer, chemotherapy, polyphenol, curcumin, quercetin, epigallocatechin gallate, resveratrol, apigenin, combination, synergism

## Abstract

This review article describes studies published over the past five years on the combination of polyphenols, which are the most studied in the field of anticancer effects (curcumin, quercetin, resveratrol, epigallocatechin gallate, and apigenin) and chemotherapeutics such as cisplatin, 5-fluorouracil, oxaliplatin, paclitaxel, etc. According to WHO data, research has been limited to five cancers with the highest morbidity rate (lung, colorectal, liver, gastric, and breast cancer). A systematic review of articles published in the past five years (from January 2018 to January 2023) was carried out with the help of all Web of Science databases and the available base of clinical studies. Based on the preclinical studies presented in this review, polyphenols can enhance drug efficacy and reduce chemoresistance through different molecular mechanisms. Considering the large number of studies, curcumin could be a molecule in future chemotherapy cocktails. One of the main problems in clinical research is related to the limited bioavailability of most polyphenols. The design of a new co-delivery system for drugs and polyphenols is essential for future clinical research. Some polyphenols work in synergy with chemotherapeutic drugs, but some polyphenols can act antagonistically, so caution is always required.

## 1. Introduction

Cancer is a generic term for a large group of diseases that can affect any part of the body. Other terms used are malignant tumors and neoplasms. One of the defining features of cancer is the rapid development of abnormal cells which grow outside of their normal limits, which can then attack neighboring parts of the body and spread to other organs, i.e., metastasize. Widely spread metastasis is the primary cause of death from cancer [1]. Cancer is the second leading cause of death in the world after cardiovascular diseases, including almost 10 million deaths in 2020 with this number expected to rise to 28.4 million by 2040 making this an increase of 47% in comparison to 2020. The most common cases of malignancies in 2020 were breast, lung, colorectal (CRC), prostate, skin (non-melanoma) and stomach cancers. However, the most common causes of cancer deaths according to World Health Organization (WHO) data were lung cancer (1.80 million deaths), colorectal (916,000 deaths), liver (830,000 deaths), stomach (gastric) (769,000 deaths) and breast (685,000 deaths) [2]. Relying on the data supplied by WHO, this study focused on preclinical and clinical research in the field of combined chemotherapy (chemotherapeutic and polyphenol), in the treatment of five types of cancer that are recognized as the leading cause of death. Lung cancer is linked to the highest morbidity and mortality in the world. Lung cancer is categorized as small-cell and non-small cell cancer (NSCLC). The latter is responsible for 85% of all lung cancers, including lung adenocarcinoma (LUADs), lung squamous cell carcinoma (LUSCs) and large cell carcinoma subtypes [3]. CRC is the third most common cancer in the world in both genders and second in the world in terms of morbidity [2]. The predictions are that the global incidence of colorectal cancer will increase to 2–5 million new cases by 2035 [4]. Environmental factors can have a great impact on the development of cancers, especially in the case of gastrointestinal cancer. Bad eating habits increase the risk of colon cancer to 70% [5], so dietary modulation, including a polyphenol-rich diet could be a strategy for preventing CRC occurrence. Hepatocellular carcinoma (HCC) is the most common type of primary liver tumor accounting for 80% of all cases of liver cancer with cholangiocarcinoma being the second most common (~10% of cases). Stomach cancer is widespread, and it is the fourth cause of cancer death in the world [2]. Breast cancer with 2.3 million new cases a year (11.7% of all cancer cases) seems to be the most widespread and fifth most lethal type of cancer in the world in 2020. Surgery, radiotherapy and systemic therapy which consists of endocrine/hormone therapy, chemotherapy, targeted therapy or a combination of these approaches is applied in breast cancer treatment [6].

Even though diagnostic tools and conventional treatment strategies are becoming more efficient every day, there are still some challenges that need to be resolved. High treatment costs and drug resistance are the main cause of treatment failure and tumor relapses, especially in the case of multidrug resistance (MDR), leading to a poor prognosis [7,8]. Overcoming chemoresistance is of the greatest importance in medical oncology. Unfortunately, chemotherapy and other types of cancer treatment can damage healthy neighboring tissue. Frequent side effects are nausea, vomiting, headaches, musculoskeletal pain, anorexia, gastritis, oral ulcers, diarrhea, constipation, alopecia and neuropathy, all of which demand additional therapies and thus a further increase in the cost of treatment [9]. Bioactive anticancer compounds originating from nature that could be combined with standard chemotherapeutics offer a possibility to overcome the side effects of chemotherapy, due to their multiple specificity, selectivity and cyto-friendly nature [10,11]. Natural compounds are non-toxic, obtainable, and rather inexpensive options when compared to the costs and time required to research and development of a completely new drug. Polyphenols are considered to be very promising anti-tumor agents. Their well-known antioxidant nature enables them to modulate tumor microenvironment (i.e., acidic pH, increased reactive oxygen species (ROS) levels, and hypoxic conditions) which can have a great impact on the emergence of drug resistance [12]. Therefore, managing the normalization of the malignant tissue microenvironment is very important. In addition to their anti-oxidative capacity which is extensively discussed in the literature [10], their biological impacts are numerous. They include anti-inflammatory, anti-cancer, anti-aging, antibacterial, and antiviral activities [13]. Due to their prebiotic role and their influence on the microbiota, the medicinal application of polyphenols is nowadays even wider [14]. Polyphenols can reduce aflatoxin-related oxidative stress and genotoxic, mutagenic, and carcinogenic effects by improving the cellular antioxidant balance, regulating signaling pathways, alleviating inflammatory responses, and modifying gene expression profiles in a dose- and time-dependent manner. Namely, aflatoxins are deadly carcinogenic mycotoxins that cause liver cancer. Flavonoids such as quercetin, oxidized tea phenols, curcumin, and resveratrol are the most studied anti-aflatoxin polyphenols [15]. 

In this review paper, we aim to provide extensive latest information (in the past five years, from January 2018 to January 2023) on combination chemotherapy with selected bioactive polyphenols, which according to all Web of Science databases, have the greatest number of scientific papers on cancer research. Due to their anticancer effect, the most researched polyphenols were curcumin (CUR), followed by quercetin (QUE), resveratrol (RES), epigallocatechin gallate (EGCG) and apigenin (AP). The effects and outcome of combined treatment of these polyphenols with chemotherapeutic drugs such as cisplatin (CIS), 5-fluorouracil (5-FU), oxaliplatin (OXA), paclitaxel (PTX), etc., in preclinical studies and clinical trials, were described. According to the WHO data, the research has been limited to five cancers with the highest morbidity rate (lung, CRC, liver, gastric and breast cancer). Due to the extensive data on the topic, all preclinical studies with polyphenols and/or chemotherapeutics applied in different types of carriers (gels, liposomes, nanotherapeutic systems) have been excluded from this review.

## 2. Discussion and Future Perspectives

Even though considerable advancements have been made in cancer treatment in the last several decades, it remains the leading cause of death worldwide. Approximately 9.6 million people die of cancer every year [2] and the estimate is that the number will be much higher in the future. This is unfortunately also true due to a reduced number of diagnostic and general physical examinations because of the redistribution of medical procedures according to priority caused by COVID-19. The medical infrastructure and staff were diverted to providing intensive care to many SARS-CoV-2 patients. At the same time, it was necessary to provide both routine and emergency medical care to all the patients, with oncology patients being particularly vulnerable [16,17]. Apart from the problems due to the pandemic, chemotherapy as a first choice in the treatment of oncology patients is increasingly limited due to the emergence of drug resistance, adverse side effects and non-selectivity during the treatment [18,19]. For that reason, new strategies are being evaluated to improve therapeutic outcomes and reduce the cost of treating malignancies, such as using natural compounds with antineoplastic properties which can protect the healthy tissue while destroying tumors without causing any additional damage [20,21].

During the last few decades, polyphenols as natural bioactive compounds and components present in food have stood out as strong chemo-sensitizing candidates with the potential to modulate numerous signaling cancer cell pathways [22,23,24,25,26]. Several polyphenols such as CUR, QUE, RSV, EGCG and AP are positioned at the very top of anticancer research. Numerous preclinical and clinical studies in addition to their antitumor and antimetastatic activities point out their favorable bioactivity including antioxidant, anti-inflammatory, antimicrobial, angiogenic, and their use in treating cardiovascular diseases, etc. [27,28,29,30].

To increase the efficiency of cancer treatment, combined therapy is used, which includes combining drugs with polyphenols that synergically interact with classic chemotherapeutics like APA, CARB, CIS, CZT, DOX, 5-FU, GEM, IRI, PTX, and others [31,32,33,34,35]. Recent preclinical research on various cancer cell lines and mice and clinical research on the deadliest types of cancer (lung, CRC, liver, stomach, and breast) were presented in this review article. The antitumor effect of polyphenols as well as their potential to synergize with chemotherapeutics is presented through data on assay type, the dosage of polyphenol and chemotherapeutic drug, and molecular mechanism, as shown in tables.

Although most studies reported positive outcomes, some negative results were observed in which, for example, some doses of CUR did not cause synergistic effects in combined therapy [36]. CUR and herceptin (HER) showed a synergistic effect on BT-474 breast cancer cells at lower concentrations of HER, but at a HER concentration of 10 µg/mL, an antagonistic effect was obtained [37]. Additionally, on HepG2 hepatocellular carcinoma cells, a CXB concentration of 100 µM showed antagonism with 40 µM CUR in contrast to lower concentrations [38]. A similar phenomenon was detected in the case of QUE where the antagonistic effect of QUE and DOX was observed in breast cancer cell lines MCF-7 and MDA-MB-231 [39]. The inconsistencies in results require further research. Careful planning of human studies is necessary due to the existence of a range of factors in the microenvironment (in vitro) in comparison to the macroenvironment and ADMET processes in vivo.

Preclinical research has paved the way for clinical studies, some of which have been completed and some are still in progress. However, the clinical therapeutic application of these combined regiments was disrupted due to major limitations of polyphenols, such as low water solubility [30,40], which results in poor bioavailability due to low absorption and low concentration of polyphenol in plasma, uneven biodistribution and poor localization in targeted anticancerogenic tissues [41]. Therefore, it is necessary to maintain a high dose of polyphenols to maintain their effective concentration levels in the blood. In practice, high doses of polyphenols could be inconvenient for oral administration (several large tablets/capsules for a single dose), and they may also cause side effects due to the irritation of the gastrointestinal tract. Even though polyphenols are an integral part of a well-balanced diet and usually are safe and easy to tolerate, the potential long-term toxicity after regular consumption of high doses needs to be addressed [42,43]. Due to the well-known nature of polyphenols, they can modulate the tumor microenvironment and affect the ROS concentrations by acting as antioxidants, or pro-oxidants [42,44,45,46]. Both antioxidant and prooxidant activity relies on the same key reactions at the molecular level [47]:

(1) ArOH+ROS→ArO•+ROSH

(2) ArO•+BM→ArOH+BM•

In the first reaction (1) phenolic compound (ArOH) reacts with reactive oxygen species (ROS) giving phenoxy radical (ArO•). In the following reaction (2), a phenoxyl radical can act as an oxidant, i.e., oxidize biomolecules, such as DNA (BM). The well-known antioxidant activity of phenolic compounds is related to the fact that a phenoxyl radical is much less reactive than ROS. Besides, the overall activity, antioxidant or pro-oxidant depends on the concentrations of the phenolic compound and formed phenoxyl radical. At low concentrations of the phenolic compound, the rate of the second reaction is low, and this reaction could be neglected, corresponding to the overall antioxidant activity. On the other side, at high concentrations, the second reaction becomes relevant and could lead to overall pro-oxidant activity. Additionally, the reactivity of phenolic compound (determines antioxidant activity) and phenoxy radical (determines pro-oxidant activity) are interconnected and influenced by the presence of substituents on the aromatic ring. For example, the presence of two or more hydroxyl groups on the aromatic ring can increase the reactivity of both the phenolic compound and phenoxyl radical, leading to greater antioxidant or pro-oxidant activity.

An improved formulation is a key step forward in the application of combined polyphenol and chemotherapy in patients. Such new and promising strategies are polyphenols in various formulations, such as nanocarriers, including polymer nanoparticles, micelles, nanoliposomes, polymer-drug conjugates, dendrimers, hydrogels, nanocapsules, and exosomes [40,48,49,50]. These formulations can ensure the effective co-delivery of polyphenols and selected chemotherapeutics into the tumor microenvironment and simultaneously reduce toxicity and increase drug stability [51,52,53]. However, despite recent advances in polymer nanoparticle therapy, scientists are still facing several challenges such as the high expenses of nanoformulations as well as their potential long-term toxicity which requires further research. Most preclinical studies in this field have shown promising results and paved the way for further in-depth clinical studies. In addition to the described polyphenols, other polyphenols such as genistein, hydroxytyrosol, oleocanthal, oleacein are the subject of clinical anticancer research [54] together with polyphenol-rich diets and plant extracts rich in polyphenols [55]. Some polyphenols obtained from “Mother Nature” work in synergy with chemotherapeutic drugs, but some polyphenols can act antagonistically, so caution is always required.

## 3. Materials and Methods

The first comprehensive database on polyphenol content in foods, Phenol-Explorer [56,57,58] and Web of Science databases (WOS) [59] were searched to collect literature for the review article. All databases, including Web of Science Core Collection, BIOSIS Citation Index, Current Contents Connect, Data Citation Index, Derwent Innovations Index, KCI-Korean Journal Database, MEDLINE, SciELO Citation Index and Zoological Record, were searched through the WOS database. During the search, filters were applied to select studies published in the last five years (from January 2018 to January 2023), while for the introduction section, description of polyphenols, and discussion with future perspectives, some older references were selected too. The databases were searched using the following term combinations: “name of polyphenol” AND one of the keywords “synergism”, “synergistic”, “interaction”, “combination”, “anticancer”, “antitumor”, “chemotherapeutic” or “chemotherapy” AND “name of the cancer” (lung, colorectal, liver, gastric (stomach), and breast cancer).

All databases in the WOS were also searched using keywords including “name of polyphenol”, “phase I (or II, II, IV)”, and “clinical trial” to collect data on clinical studies of the synergistic anticancer mechanism of polyphenols and chemotherapy. Clinical study databases such as ClinicalTrials.gov [54] were also searched.

## 4. Curcumin

CUR is the main natural polyphenol that is found in the rhizome of *Curcuma longa* (up to ~5%). It is a lipophilic compound, insoluble in water and in acidic and neutral solutions, but soluble in ethanol, dimethylsulfoxide and acetone. Because of its intense yellow color, it is used as a natural food coloring agent, and it has been assigned an E number (E100). It has been used for centuries in both Ayurvedic and traditional Chinese medicine [60]. In addition to this traditional use, it has various other effects, including antioxidant and antiproliferative and anti-aging, it is also used in treating Parkinson’s and Alzheimer’s diseases, diabetes, and cardiovascular diseases [41,61]. It has antitumor effects on diverse types of cancer, including breast cancer, CRC, liver cancer, glioblastoma, gastric cancer, lung cancer, etc. [60,61]. The antitumor effect of CUR is aimed at several signal pathways included in the regulation of cell proliferation, invasion, metastasis, and apoptosis [41]. Despite distinct functions, it has limited application due to low water solubility leading to low absorption and low oral bioavailability [62].

Researchers tried to change these adverse effects by screening CUR analogs, by using piperine that interferes with glucuronidation, producing liposomal CUR or polymeric CUR nanoparticles [60,63].

Numerous studies have investigated CUR as a possible natural agent in lung cancer therapy. At the end of the 20th century, researchers discovered that CUR could suppress lung tumor metastasis and extend the lifespan of mice. There are different modes and pathways of action of CUR on non-small cell lung cancer (NSCLC). The results of preclinical studies show that CUR can inhibit tumor nodules [64], control the cell cycle [65], induce ROS production and endoplasmic reticulum (ER) stress [66], suppress the migration of cancer cells [67,68,69], trigger apoptosis [70,71], increase DNA damage and ferroptosis [69,72]. CUR is also effective in restraining cancer stem cells [73] and promotes necrotic cell death [74]. Many studies have reported that CUR is a perfect adjunctive agent because it increases the sensitivity of NSCLC to some chemotherapy drugs. By regulating different mechanisms, CUR acts synergistically with chemotherapeutics to slow down the growth of NSCLC. In some cases, it also reduces the toxicity of chemotherapeutics.

The anticancer effect of CUR in CRC may be mediated by several mechanisms, resulting in reduced cell growth and increased apoptosis. CUR stimulates the production of ROS and Ca^2+^ and induces caspase-3 activity [75]. Besides, CUR inhibits the cell cycle, activates p53 (only in p53^+/+^ cells) and p21 [76], and induces cellular senescence (irreversible growth arrest of proliferating cells) by activating the lysosomal senescence enzyme associated-β-galactosidase (SA-β-gal) and by upregulating p21 protein [76,77]. In addition, CUR-induced apoptosis is associated with oxidative stress caused by superoxide anion production, which contributes to p53-independent cellular cytotoxicity [78]. As a plant polyphenol, CUR has been shown to have the ability to alleviate the resistance of CRC to chemotherapeutic agents with still unclear mechanisms. Several preclinical studies demonstrated the improvement in the therapeutic effectiveness of CRC cells when these chemotherapeutic agents are co-administered with CUR.

CUR has a potential role in treating liver cancer [79]. For example, CUR inhibits HCC metastasis and invasion by inhibiting microRNA-21 expression [67] and inhibits HCC proliferation by reducing VEG expression [80]. Different combinations of CUR with an anticancer drug were also investigated.

Moreover, evidence shows that CUR has a significant inhibitory effect on the proliferation of gastric cancer cells by targeting various cancer-related signaling pathways, such as apoptosis [81].

CUR exerts an anti-breast cancer impact by targeting various regulatory proteins, including those of kinases, transcription factors, receptors, enzymes, growth factors, cell cycle, and apoptosis-related molecules, as well as microRNAs. It has also been shown to modulate a variety of key signaling pathways of JAK/STAT, NF-kB, Wnt/β-catenin, PI3K/Akt/mTOR, MAPK, apoptosis, and cell cycle pathways involved in breast cancer progression and development [82].

### 4.1. Curcumin Combined with Chemotherapy in Preclinical Studies

#### 4.1.1. Lung Cancer

Researchers investigated the effectiveness of CUR as a chemosensitizer in a subpopulation of cancer stem cells (CSCs) of NSCLC. CIS alone or combined with CUR was administered to lung cancer adenocarcinoma cells A549 and H2170 over 24, 48, and 72 h. The results showed that CUR combined with CIS effectively inhibits the self-renewal ability of CSCs and prevents drug resistance [83]. In another study, the combination of CUR and CIS improved the sensitivity of A549 cells to X-rays, reducing cancer growth most likely by blocking Epidermal Growth Factor Receptor (EGFR)-related signaling pathways [84]. Co-treatment with CUR and CIS also suppresses A549 cell survival and mediates apoptosis by targeting Cu-Sp1-CTR1 [85].

Targeted cancer drugs such as crizotinib (CZT) and gefitinib (GEF) are used in the treatment of NSCLC with gene mutations; however, the development of drug resistance is possible. The research showed that the combination of CUR and CZT upregulates the expression of miR-142-5p to target Ulk1 and inhibits autophagy in NSCLC cells. In this way, CUR reduces the resistance of lung cancer cells to the drug CZT [86]. Co-treatment with CUR and GEF promotes autophagy and autophagy-mediated apoptosis in resistant NSCLC cells. Combined therapy significantly inactivates EGFR by retarding Sp1, influencing the interaction between Sp1 and HDAC1 [87]. These findings indicate that CUR and targeted agents may work together to provide effective therapy for advanced NSCLC.

Common organic drugs for lung cancer chemotherapy, such as gemcitabine (GEM), are also being investigated in combination with numerous bioactive natural products, including CUR, due to the emergence of resistance in patients. Namely, for cells resistant to GEM, the simultaneous administration of CUR and GEM does not increase toxicity in mice, and it dramatically increases the sensitivity of resistant cells to GEM. Combination treatment of CUR and GEM inhibited invasion and migration in GEM-resistant lung cancer cells through downregulation of MMP9, vimentin, and N-cadherin and overexpression of E-cadherin [88].

**Figure 1:**
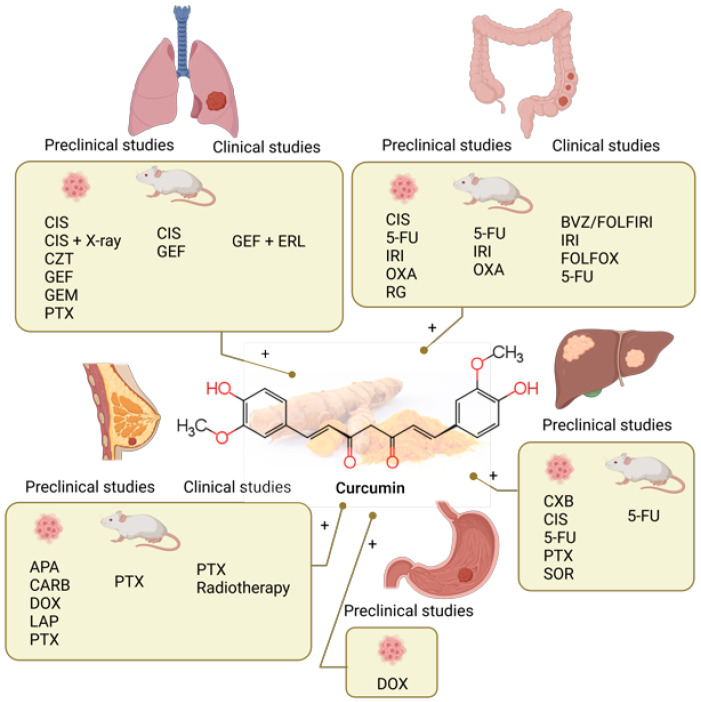
Combination of curcumin and chemotherapy in preclinical (lung [83,84,85,86,87,88,89], colorectal [90,91,92,93,94,95,96,97,98,99,100], liver [38,101,102,103,104], gastric [81] and breast cancer [105,106,107,108,109,110,111]) and clinical (lung [112], colorectal [113,114,115,116,117,118,119] and breast cancer [120,121,122]) studies in the past five years. CIS: cisplatin; CZT: crizotinib; GEF: gefitinib; GEM: gemcitabine; PTX: paclitaxel; 5-FU: 5-fluorouracil; IRI: irinotecan; OXA: oxaliplatin; RG: regorafenib; CXB: celecoxib; SOR: so-rafenib; DOX: doxorubicin; APA: apatinib; CARB: carboplatin; LAP: lapatinib. Created with BioRender.com.

The study by Lee et al. reported that a simple dry powder inhalation formulation of a CUR and PTX exhibits a more potent cytotoxic effect against lung cancer cells. This effect is evident from the induction of apoptosis/necrotic cells and G2/M cycle arrest in A549 and Calu-3 cells. Increased intracellular ROS, mitochondrial depolarization, and reduced ATP content in A549 and Calu-3 cells showed that the effect of the combination of CUR and PTX is related to mitochondrial oxidative stress. Interestingly, the presence of CUR is crucial for neutralizing the cytotoxic effects of PTX on healthy cells (Beas-2B) [89] (Table 1, Figure 1).

#### 4.1.2. Colorectal Cancer

CIS is one of the most frequently used chemotherapy drugs for diverse types of cancer, including CRC. Although chemotherapeutic strategies have improved the patient prognosis and survival rate, developing resistance to CIS leads to relapse. In their study, Fan et al. showed that CUR acts synergistically with CIS and suppresses the proliferation of CIS-resistant colon cancer cells (HT-29). Glutamine metabolism in cancer cells was markedly elevated, displaying a glutamine-dependent phenotype. It has been concluded that CUR could also be applied clinically against CRC by modulating glutamine metabolism inhibited by miR-137 [90].

There are several studies examining combinations of CUR and 5-FU. Research on cell lines SW480 and HT-29 showed that combinations of low doses of CUR with 5-FU also reduce cell resistance to 5-FU. The authors reported on G2/M phase cell cycle arrest and downregulation of NNMT by p-STAT3 depression [91]. Another study recorded a significant reduction in the proliferation and migration of SW620 cells in female nude mice. A significantly increased apoptosis rate prolonged the survival of immunodeficient mice in the combination group compared to that in the 5-FU group. The results showed that CUR significantly inhibits pERK signaling and reduces L1 expression in SW620 cells [92]. A study by Lu et al. showed that increasing the concentration of CUR increases the sensitivity of HCT-116 cells resistant to 5-FU. CUR contributed to the inhibition of proliferation, induction of apoptosis and block of the G0/G1 phase on 5-FU treated HCT-116 cells. WNT signaling pathway and epithelial-mesenchymal transition (EMT) progress was slowed by significantly inhibited TET1 and NKD2 expression. In addition to Pax-6, TET1 and NKD2, CUR inhibits the WNT signal pathway and EMT progress [93]. CUR can reverse effects on the MDR of human colon cancer cell lines HCT-8/5-FU by downregulation of P-gp and HSP-27 [94].

Irinotecan (IRI) in combination with CUR had synergistic antitumor effects in CT-26 colon carcinoma cells. Combination treatment significantly upregulated ICD-related proteins, including CALR and HMGB1, and had a more significant antitumor effect than IRI or CUR single therapy in vivo. Combination treatment promotes the tumor immune response and prolongs the tumor-free time in mice [95]. CUR has exerted a protective effect against IRI drug-induced intestinal mucosal injury. The protective effect is mediated by the inhibiting NF-κB activation, oxidative stress, and ER stress induced by IRI [96]. Promising data are available regarding the re-sensitization of IRI-resistant cells. Su et al. showed that CUR could effectively reduce the chemoresistance of CRC cells by inducing apoptosis in the IRI-resistant cells. CUR significantly alters the expression levels of CSC identification markers. Moreover, CUR upregulated the expression of Bax pro-apoptotic protein while downregulated anti-apoptotic Bcl-2 [97]. Zhang et al. in their study showed that CUR is an effective chemo-sensitizing agent that can reverse EMT in CRC. IRI-resistant CRC cells (LoVo/CPT-11R) treated with CUR have upregulated E-cadherin expression, while vimentin and N-cadherin expressions have been downregulated [98].

Combined treatment with CUR and the chemotherapy drug OXA improves the therapeutic efficacy of the drug. Apoptotic activity was enhanced, and growth inhibition of CRC increased. One study confirmed the result in vivo using HCT116/OXA xenograft mice, showing that tumor volume, and weight and Smad2/3 levels were reduced when animals were treated with combination regimens compared to those treated with OXA alone [99].

CUR enhanced the growth inhibition in human CRC cancer HCT 116 cells (KRAS mutant) to a greater extent than in human CRC HT-29 cells (KRAS wild-type). Flow cytometric analysis showed that adding CUR elevated apoptosis and significantly increased autophagy in HCT 116 but not in HT-29 cells. Mechanistically, CUR behaved like a MEK-specific inhibitor (U0126). The potential role of CUR in regorafenib (RG)-treated KRAS mutant CRC cancer is indicated by the fact that CUR may target one additional gene other than mutant KRAS [100] (Table 1, Figure 1).

#### 4.1.3. Liver Cancer

To maintain the anticancer effect of celecoxib (CXB) with a minimal toxicity profile, a low concentration of the drug was combined with CUR. The combined administration synergistically induced apoptosis in liver cancer cells, leading to an increase in caspase-3 activity. Cell proliferation analysis revealed that HCC HepG2 cells showed a significant decrease in the expression of cell survival proteins, such as Akt, NF-κB p65 and malondialdehyde (MDA), and the inhibition of VEGF expression. Simultaneous treatment with CUR and CXB indicated the strengthening of antiproliferative and anti-angiogenic effects [38].

The cytotoxic effects of CIS in combination with CUR were investigated in various cell lines, including human HCC HepG2 cells. The results showed no adverse interactions between CUR and CIS regarding cell viability. The combination of CUR and CIS could be a helpful therapeutic approach for the treatment of human cervical cancer and HCC [101].

CUR also limits DOX-mediated cardiotoxicity through modulation of altered calcium flux, mitochondrial damage, oxidative stress, and initiation of apoptosis in cardiac tissue. In cells treated with DOX, CUR decreased the expression of the cardiotoxic marker SCK and increased the expression of superoxide dismutase (SOD) and catalase (CAT) [123].

HCC cell lines and mice were used to investigate the synergistic effects of CUR and 5-FU. The cytotoxicity test results showed that in comparison to the use of individual drugs, the combination of CUR and 5-FU (1:1, 1:2, 1:4, 2:1 and 4:1, mol/mol) demonstrated more potent cytotoxicity in SMMC cells -7721, Bel-7402, HepG-2 and MHCC97H. Among them, the combined group molar ratio of 2:1 showed a strong synergistic effect in SMMC-7721 cells. The mechanism of the synergistic effect may be related to the inhibition of NF-κB (overall) and COX-2 protein expression. In addition, the synergistic effect was also confirmed in xenograft mice in vivo [102]. 

The synergistic efficacy of CUR and PTX in Hep3B and HepG2 hepatoma cells has been demonstrated through the downregulation of Lin28. Lin28B silencing reduced the chemoresistance of PTX-resistant HCC cells [103]. 

Considerable efforts have been made to improve the therapeutic efficacy and reduce the side effects of sorafenibe (SOR). Thus, the aim of Bahman’s study from 2018 was to investigate whether the combined therapy with natural phenolic compounds, including CUR, would reduce the dose of SOR without a concomitant loss of its effectiveness. Concomitant treatment with SOR and CUR caused S phase and G2/M phase arrest of liver cancer cells and markedly induced apoptosis. Furthermore, concomitant treatment with SOR and CUR reduced the protein levels of cyclins A, B2 and D1, phosphorylated retinoblastoma and B cell lymphoma (Bcl) extra-large protein. By contrast, SOR and SOR co-treatment increased the protein levels of Bcl 2 associated X protein, cleaved caspase-3, and cleaved caspase-9 in a dose-dependent manner. It was concluded that when combined with SOR, CUR augmented the apoptosis-inducing potential of SOR [104] (Table 1, Figure 1).

#### 4.1.4. Stomach Cancer

DOX hydrochloride is one of the most important chemotherapy agents against cancer, with limited therapeutic efficacy in the treatment of GC. Therefore, exploiting synergistic effects with strategies such as combination therapy seems appropriate and promising in treating GC. Thus, CUR and DOX co-treatment showed a significantly greater induction of apoptosis and anti-mobility behavior of AGS GC cells when compared to monotherapy and the untreated control [81] (Table 1, Figure 1).

#### 4.1.5. Breast Cancer

The study showed that CUR and apatinib (APA) inhibit the growth and proliferation of breast cancer cells by inducing the apoptotic pathway and regulating the expression of apoptosis-related genes. The combination of CUR and APA induces breast cancer cell apoptosis by increasing the expression of the apoptosis-inducing BAX and SMAC genes. There is also a decrease in the expression of the apoptosis inhibitor BCL2 and the SURVIVIN gene [105].

As patients with triple-negative breast cancer (TNBC) have a feeble response to hormone inhibition or anti-HER2 therapy, traditional chemotherapy is commonly used in these patients. Recently, carboplatin (CARB) has been approved for the clinical treatment of TNBC. However, some patients exhibited resistance to CARB treatment. To improve the sensitivity of resistant TNBC cells to CARB, the treatment of cancer cells with CUR and CARB was applied. The combination was found to inhibit proliferation and induce apoptosis. Mechanistically, CUR exerted its anticancer effect by increasing the production of ROS. This reduced the DNA repair protein RAD51, which led to the upregulation of γH2AX. As expected, the ROS scavenger NAC reversed the CUR-mediated growth inhibitory effect and DNA repair pathway activity [106].

The second study was designed to evaluate the underlying mechanisms of Aurora A mediated DOX insensitivity in MCF-7Dox/R, an isolated resistant subline of the MCF-7 cancer cell line. The study concludes that molecular targeting of Aurora A by CUR restores chemosensitivity by increasing the efficacy of DOX in breast cancer [107]. The previous study showed that the combined treatment of CUR and DOX decreased the IC_50_ value of the drug. It also increased the sensitivity of DOX-resistant MCF-7 and MDA-MB-231 cells via the inhibition of ABCB4 activity. This effect, which is mediated by inhibition of the ATPase activity of ABCB4 without altering protein expression, leads to increased intracellular levels of DOX. The above can help treat drug-resistant breast cancer cells [108].

Preclinical studies in animal models of TNBC pointed out the key role of thymidylate synthase in the regulation of the synergism of CUR and 5-FU. The study also confirmed the pharmacological safety of the CUR and 5-FU combination using an acute and chronic toxicity study in Swiss albino mice [124].

CUR can enhance the effectiveness of lapatinib (LAP) in treating Her2-dependent breast cancer [109].

A recent report suggests that cotreatment of CUR and PTX inhibited aldehyde dehydrogenase-1 (ALDH-1) and PTX-induced Pgp-1 expression in MCF-7 cells. This study has also demonstrated the synergistic cytotoxic interaction of the CUR-PTX combination accompanied by upregulation of Bax, caspase-7, and caspase-9, along with downregulation of Bcl-2 expression in treated cells. Besides, in vivo animal experiments on Ehrlich ascites carcinoma (EAC)-tumor-bearing mice also showed a reduction in tumor size and marked inhibition of PTX-induced Pgp-1 and ALDH-1 protein expression in tumor tissue [110]. A similar drug synergism between CUR and PTX showed antitumor efficacy via regulation of P-glycoprotein and ALDH-1 in MCF-7 breast cancer-bearing mice [111] (Table 1, Figure 1).

### 4.2. Clinical Studies of Curcumin Combined with Chemotherapy

Numerous clinical trials of the combination of CUR and anticancer drugs have been conducted despite the low bioavailability of CUR, so in some studies, unique formulations such as liposomal CUR were used. CUR has been tested in clinical studies in various malignant diseases; CRC, breast, pancreatic cancer, haematological malignancies, etc. [11]. Combined therapy with CUR has been proven safe and tolerable in clinical trials of breast cancer, chronic myeloid leukaemia, CRC, pancreatic cancer, and prostate cancer [36]. The latest clinical studies on the treatment of CRC deal with the combinations of CUR with 5-FU and IRI [113], IRI [114] and FOLFOX therapy [115]. The completed study of combined CUR and PTX treatment of breast cancer resulted in fewer toxic side effects of chemotherapy and improved quality of life [120] (Figure 1).

An interventional clinical trial (NCT02439385) [116] with 44 participants had the primary objective to evaluate progression-free survival in patients with colon cancer with inoperable metastases after first-line treatment with Bevacizumab (BVZ)/FOLFIRI (folinic acid, bolus/continuous 5-FU, and IRI) in combination with a dietary supplement of nanostructured lipid particles containing CUR. During treatment, patients received an i.v. drug every 14 days and daily as a dietary supplement, nanostructured lipid particles of CUR in a dose of 100 mg. Combined therapy had acceptable safety and tolerability with comparable long-term survival rates, although the authors state that additional randomized controlled trials are still needed [113] (Figure 1).

A prospective evaluation of the effect of CUR (NCT01859858) on the toxicity and pharmacokinetics of IRI was investigated in patients with colon cancer [117]. It was concluded that up to 4 g of phosphatidylcholine CUR (PC), the formulation could be safely administered with IRI without impacting the pharmacokinetic and adverse event profile of IRI [114] (Figure 1).

In another study (NCT01490996), ref. [118] the safety and tolerability of CUR (up to 2 g) was documented when administered together with combination chemotherapy that was consisting of folic acid, 5-FU and OXA (FOLFOX) in patients with metastatic CRC. The results of phase I/IIa studies showed that such combined therapy showed a higher objective response rate (ORR) with more prolonged median progression-free survival (PFS) and overall survival (OS) compared to the exact parameters of patients treated with FOLFOX chemotherapy alone [115] (Figure 1).

In completed clinical trials on breast cancer, a double-blind, randomized, phase II clinical trial (NCT03072992), was performed [121]. The primary objective of the trial was to evaluate the efficacy of combined therapy with CUR and PTX versus PTX in patients with advanced and metastatic breast cancer. The results showed that treatment with CUR in combination with PTX was better than the combination of PTX and placebo in terms of ORR and physical performance after 12 weeks of treatment. Intravenously administered CUR did not cause significant safety problems or reduce the quality of life [120] (Figure 1).

Studies that are still ongoing or the results of which have not yet been published refer to lung carcinoma, CRC, and breast cancer. A Phase 1 Open-label Prospective Cohort Trial of CUR Plus Tyrosine Kinase Inhibitors for Epidermal Growth Factor Receptor (EGFR)-Mutant Advanced Non-small Cell Lung Cancer is the title of a preliminary clinical trial (NCT02321293) [112] in which patients daily receive the drugs GEF and ERL and Longvida^®^ Optimized CUR for eight weeks. The following study (NCT02724202) aims to confirm clinical safety and identify the clinical response rate of combination treatment with CUR and 5-FU in chemo-refractory CRC patients. All subjects (13 participants) will receive induction oral CUR 500 mg twice daily for two weeks. Patients will continue to receive CUR at the same dose for an additional six weeks while being treated with three cycles of 5-FU [119] (Figure 1).

Phase II Study of CUR vs. Placebo for Chemotherapy-Treated Breast Cancer Patients Undergoing Radiotherapy (NCT01740323) is a completed study with unpublished results. The primary purpose of the investigation is to determine if CUR reduces NF-kB DNA binding and, ultimately, its downstream mediator IL-6 in patients receiving XRT for their breast cancer after having completed chemotherapy [122] (Figure 1).

## 5. Quercetin

Quercetin (QUE), a natural flavonoid present in many plants, especially in red onion, citrus fruits, green leafy vegetables, broccoli, apples, berries, green tea and coffee shows a wide range of pharmacological activities such as antioxidative, anti-inflammatory, anticancer, analgesic, neuroprotective, cardioprotective, hepatoprotective, nephroprotective, otoprotective, antibacterial (*M. tuberculosis*), antiviral (COVID-19), immunomodulatory, and anti-allergy activities. These activities lead to beneficial effects in various diseases: cancer, diabetes, obesity, hyperuricemia, neurodegenerative diseases, cardiovascular disease, pulmonary disorders, and osteoporosis and it shows anti-aging effects [40,125,126]. QUE exerts beneficial effects in several types of cancer, such as breast, cervical, ovary, endometrial, prostate, gastric, hepatocellular, pancreatic, colorectal, oral, lung, senescence-mediated cancer, leukemia, acute myeloid leukemia, Burkitt’s lymphoma and lymphoma [127]. It shows a biphasic, dose-dependent anticancer effect. At low concentrations, QUE acts as an antioxidant with chemo-preventive effects, while at high concentrations it acts as a prooxidant showing chemotherapeutic effects. QUE modulates the activity of signaling pathways and expression of miRNAs related to anticancer and anti-inflammatory effects. It reduces proliferation, induces apoptosis, arrests the cell cycle, induces autophagy, prevents cancer metastasis, and inhibits angiogenesis [44]. One of the key features of QUE is its pro-apoptotic effect which it exerts by inhibiting the Akt and NF-*κ*B signaling pathways. It induces the downregulation of anti-apoptotic Bcl-2 and upregulation of pro-apoptotic Bax, and increases cytochrome C levels and cleaved forms of caspase-9, caspase-3, and PARP-1 [128]. QUE reduces proliferation by inhibiting intracellular signaling pathways such as PI3K, EGFR, and Her2/neu. It regulates and inhibits the cell cycle by activating p21, arrests the cell cycle at the G1 phase and inhibits microtubule polymerization which also affects the cell cycle. QUE attenuates the progression of colon cancer through cell cycle arrest, decreased cell viability, induction of apoptosis and autophagy, and inhibition of metastasis. In colon cancer cells, QUE induces apoptosis, by activating the MPAK signaling pathway, and by downregulating the Wnt/β-catenin pathway and related genes. It reduces cell migration by the suppression of MMP-2 and MMP-4. Additionally, QUE inhibits colorectal cell lung metastasis. Other relevant pathways are P13K/AKT/mTOR, JNK/JUN and NF-*κ*B [129]. The effect on gastric cancer is related to cell cycle arrest and promotion of apoptosis, and the inhibition of the growth of gastric cancer stem cells by inducing mitochondrial-dependent apoptosis. In human breast cancer cells, QUE causes cell cycle arrest, induces apoptosis, and inhibits proliferation. It exhibits dose-dependent estrogenic and anti-estrogenic properties [126]. QUE induces p53 expression in MDA-MB-453 and human basal-like MDA-MB-468 breast cancer cells. It affects the G1 phase and induces apoptosis by suppressing cyclin D1, P21 and Twist expression in MCF-7 cells, by the P38MAPK pathway. QUE inhibits cell proliferation by the modulation of PI3k, EGFR, and Her2/neu and increases the expression of pro-apoptotic proteins including Bax and Bak, while it decreases Bcl-2 expression. The bioavailability of QUE is low, about 2% after oral administration, which is related to low solubility and gastrointestinal instability. Approximately 90% of QUE administered through the intraperitoneal route is metabolized after one hour. Currently, many studies are oriented toward the development of different drug delivery systems, such as QUE-loaded nanocarriers, nanoparticles, polymeric micelles, conjugates, inclusion complexes, and nanosuspensions [40].

### 5.1. Quercetin Combined with Chemotherapy in Preclinical Studies

#### 5.1.1. Colorectal Cancer

**Figure 2:**
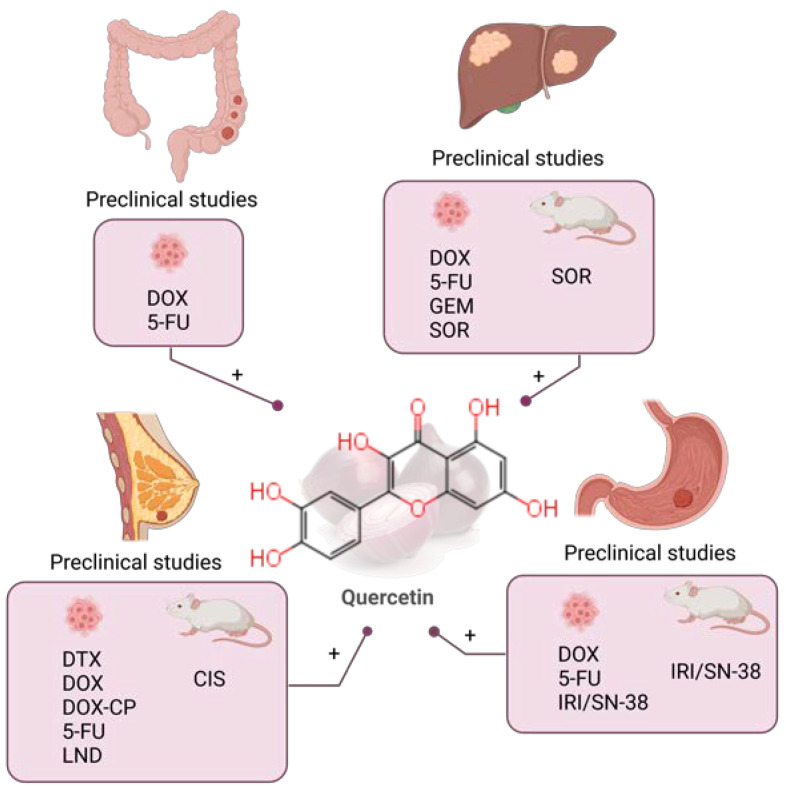
Combination of quercetin and chemotherapy in preclinical (colorectal [130,131,132], liver [133,134,135,136], gastric [137,138,139] and breast cancer [39,140,141,142,143,144,145,146]) studies in the past five years. DOX: doxorubicin; 5-FU: 5-fluorouracil; GEM: gemcitabine; SOR: sorafenib; IRI: irinotecan; CIS: cisplatin; DTX: docetaxel; CP: cyclophosphamide; LND: lonidamine. Created with BioRender.com.

One of the primary mechanisms of MDR is the overexpression of ATP-binding cassette (ABC) efflux transporters, such as P-glycoprotein, and it is known that QUE is able to inhibit P-gp mediated MDR in various cancer cells. Zhou et al. studied the effect of QUE alone and in combination with DOX on SW620/Ad300 cells (P-gp-overexpressing Dox-resistant cells) [130]. QUE enhances the sensitivity of colon cancer cells SW620/Ad300 cells to DOX. Both treatments with QUE alone and in combination with DOX inhibit the upregulated glutamine metabolism in DOX-resistant cells by inhibiting the expression of the glutamine transporter solute carrier family 1, member 5, SLC1A5. Additionally, QUE downregulates the glutamine metabolism-dependent TCA cycle, which downregulates the ATP level and inhibits the ATP-driven efflux activity of P-gp. In this way, it increases the intracellular accumulation of DOX and enhances the sensitivity of SW620/Ad300 cells to DOX. QUE enhances the inhibitory activity of DOX on DNA replication and transcription and reduces the level of GSH, which restores the sensitivity of SW620/Ad300 cells to oxidative stress. 5-FU is the most widely used chemotherapeutic drug in treating CRC. However, its toxicity to normal tissues and resistance limits its use. Earlier studies have shown that QUE increases the activity of 5-FU by inducing apoptosis in CRC cells with the wild-type p53 gene. Erdogan et al. studied the combined treatment with QUE and 5-FU in HT-29 human colorectal adenocarcinoma cells [131]. The results of this study showed that QUE alone and in combinations with 5-FU inhibited the growth of HT 29 cells with the combination index (CI) value of 0.5, indicating synergistic effects. QUE used alone induced a lower rate of apoptosis compared with 5-FU alone by increasing p53, Bcl-2 and Bax expression levels. IC_50_ dose of 5-FU, QUE, and 5-FU + QUE enhanced the apoptosis by 5.2, 4.5, and 8.1-fold compared to the control, respectively. Combined treatment synergistically reduced the Akt/mTOR protein and reduced VEGF and angiogenesis. Terana et al. studied the anti-tumor effect of QUE + 5-FU in HCT-116 and Caco-2 cells [132]. The combination showed higher cytotoxic effects compared with 5-FU alone. It enhanced apoptosis and inhibited the expression of miR-27a, leading to the upregulation of secreted frizzled-related protein 1 and suppression of Wnt/β-catenin signalling, which is one of the main dysregulated pathways in CRC (Table 2, Figure 2).

**Table 2:** Table summarizing the combination therapy in the past five years of quercetin with chemotherapy in the preclinical studies in vitro (cell lines) and in vivo (rodents).

Cancer Type	Chemotherapy	Dosage	Assay Type	Molecular Effect	Study Conclusion	Ref.
Colorectal cancer	DOX	33 µM QUE + 0.5 µM DOX	SW620/DOX drug-resistant cell line and SW620/Ad300 cell line	Reversed P-gp-mediated drug resistance, increased intracellular DOX accumulation; modulates glutamine metabolism in DOX-resistant cells by inhibition of SLC1A5.	Reversed MDR, enhanced sensitivity to DOX.	[130]
	5-FU	180 µg/mL QUE + 110 µg/mL 5-FU	HT-29 cell line	Decreased angiogenesis by inbibition of VEGF.	Synergistically enhanced the anticancer effect of 5-FU.	[131]
	5-FU	12 µg/mL QUE + 62.5, 125 µg/mL 5-FU	HCT-116 cell line	Enhanced apoptosis; suppression of Wnt/β-catenin signalling.	Enhanced 5-FU sensitivity.	[132]
Liver cancer	DOX, 5-FU	40–160 µM QUE + 0.2–125 µg/mL DOX/5-FU	BEL-7402 and BEL-7402/5-FU drug-resistant cell lines	Inhibition of FZD7/β-catenin pathway and ABCB1, ABCC1 and ABCC2 efflux pump.	Enhanced DOX and 5-FU sensitivity.	[133]
	DOX	0–100 μM QUE + 5–50 μM DOX	HepG2 cell line	Suppresses the efflux activity of MDR1, downregulates HIF-1α; increases apoptosis rate, upregulates p53 and cleaved caspase 3.	Enhances cytotoxic activity of DOX.	[134]
	GEM	100–200 μM QUE + 38 mg/mL GEM	HepG2 cell line	Promotes apoptosis, induces S phase cycle cell arrest by upregulation of p53 and downregulation of cyclin D1.	Increases anticancer effect.	[135]
	SOR	In vitro: QUE 20–220 uM; SOR: 5–40 uM); SOR + QUE = 1:1:6.25 uM; 12.5 uM; 25 uM; 50 µM i 100 µM. In vivo: 7.5 mg/kg/day SOR, 2 h later 50 mg/kg/day QUE	HepG2 cell line and chemically induced HCC rat model	Suppressed proliferation, enhanced apoptosis and necrosis.	Synergistically increases anticancer effect and increases liver recovery.	[136]
Gastric cancer	DOX	100–200 μM QUE + 0.25–1.25 μM DOX	KATO III cell line	Enhanced apoptosis; upregulation of γH2As.	Increases chemotherapeutic effects.	[137]
	5-FU; DOX	50 μM QUE + 25 μM 5-FU; 50 μM QUE + 0.5 μM DOX	AGS-cyr61 cell line	Reverses multidrug resistance; decreased CYR61, MRP1, and p65; induced caspase-dependent apoptosis; suppressed migration and down-regulation of EMT-related proteins; inhibits colony formations.	Strong synergistic effects with 5-FU and DOX.	[138]
	IRI/SN-38	In vitro: 12.5, 50 μM QUE + 5, 25 nM SN-38. In vivo: 20 mg/kg i.p. injection	AGS-cyr61 cell line and AGS xenograft mouse model	In vitro: induces apoptosis, decreases cancer cell metastasis, downregulates β-catenin. In vivo: modulation of angiogenesis-associated and EMT-related factors.	Enhances cytotoxic effects of IRI/SN-38.	[139]
Breast cancer	CIS	30 mg/kg QUE + 7 mg/kg CIS	Breast tumor-bearing mouse model	Inhibited tumor growth and reduced renal toxicity.	Synergistic effect; inhibits renal toxicity induced by CIS.	[140]
	DTX	95 μM QUE + 7 nM DTX	MDA-MB-231 cell line	Inhibited cancer cell growth, induced apoptosis.	Enhances cytotoxic effects of DTX, decreases toxic effects.	[141]
	DOX	0.7 μM QUE + 2 μg/mL DOX	MCF-10A, MCF-7 and MDA-MB-231 cell lines	Increased intracellular accumulation of DOX in cancer cells by downregulating the expression of P-gp, BCRP and MRP1; decreased cytotoxicity of DOX to non-tumoral MCF-10A mammary cells and myocardial AC16 cells.	Increases chemotherapeutic effects of DOX at a lower concentration; decreases the toxic side effects of DOX.	[142]
		98 μM QUE + 0.35 μM DOX for MCF7 cells; 38 μM QUE + 0.35 μM DOX for MDA-MB-231 cells; 78 μM QUE + 0.35 μM DOX for T47D cells	MCF-7, MDA-MB-231 and T47D cell lines	Modulates vasoconstriction/vasodilatation induced by DOX; inhibited ROS generation; interferes DOX-induced cell cycle arrest; enhances intracellular concentration of doxorubicin in MDA-MB-231 and T47D cells by inhibition of P-gp.	Decreases cardiotoxicity; strong antagonistic interaction in MCF-7 and MDA-MB-231 cells.	[39]
	DOX-CP	20 μM QUE + (0.5 μg/mL DOX + 40 μg/mL CP); 1–40 μM QUE + (0.5 μg/mL DOX + 40 μg/mL CP)	MDA-MB-231 cell lines	Reduces cardiotoxicity by activating ERK1/2 pathway in cardiomyocytes; enhances the antitumor activity of DOX-CP by inhibiting ERK1/2 pathway in TNBC cells.	Enhances chemotherapeutic effects of DOX-CP; decreases DOX-CP induced cardiotoxicity.	[143]
	5-FU	150, 300, 446 μM QUE + 100 μM 5-FU	MCF-7 cell line	Enhanced apoptosis by increased expression of Bax and p53 and caspase-9 activity and decreasing the Bcl2 expression; decreased colony formation.	Enhances the sensitivity of breast cancer to 5-FU.	[144]
		50, 200 μM QUE + 1.5, 6.25, 25 μM 5-FU	MDA-MB-231 cell line	Decreased migration rate and MMP-2 and MMP-9 gene expressions.	Synergistic effect.	[145]
	LND	80 μM QUE + 0.1, 1, 5 μM LND	MCF-7 cell line	Induced cell cycle arrest in the G2/M phase, arrested the cell cycle at S point; induced apoptosis by increased caspase levels, decreased MMP-2/-9 mRNA expression.	Synergistic effect.	[146]

QUE: quercetin; DOX: doxorubicin; 5-FU: 5-fluorouracil; GEM: gemcitabine; SOR: sorafenib; IRI: irinotecan; CIS: cisplatin; DTX: docetaxel; CP: cyclophosphamide; LND: lonidamine.

#### 5.1.2. Liver Cancer

MDR is the critical limitation for the treatment of liver cancer. Chen et al. investigated the effect of QUE on MDR on HCC multidrug resistant BEL-7402/5-FU (BEL/5-FU) cells [133]. The treatment with QUE increased the accumulation of DOX. QUE enhanced the chemosensitivity of BEL/5-FU cells to 5-FU by 1.63–3.41-fold and by 1.36–2.51-fold to DOX. QUE inhibited the functions and expressions of ABCB1, ABCC1 and ABCC2 efflux pump by the downregulation of ABCB1, ABCC1 and ABCC2, through the FZD7/Wnt/β-catenin pathway. Hassan et al. investigated the combined treatment of QUE and DOX on human HCC cell line HepG2 in 2D and 3D cultures [134]. The efficiency of the drug is always lower in 3D than in 2D culture, which is related to a decreased accessibility of the drug to its target cell in the 3D environment. Administration of both DOX and QUE alone showed only a mild effect on the apoptosis rate, while combined treatment with DOX (10 μM) and QUE (50 μM) was much more effective both in 2D and 3D cultures. Additionally, they found that QUE used alone or in combination with DOX can suppress the efflux activity of MDR1 in HepG2 cells. The combination downregulates HIF-1α, which is strongly expressed in 3D culture. Interestingly, HepG2 cells cultured in 2D monolayer conditions do not express HIF-1α.

QUE inhibits the proliferation and apoptosis of GEM-resistant cell lines [135]. QUE led to the accumulation of cells in S phase, with a concomitant decrease in the G1 and G2/M phase populations, by the upregulation of tumor protein p53 and the downregulation of cyclin D1. The effect differs in combinations with different concentrations of QUE. The combined treatment with a low QUE concentration (100 μM) significantly decreased S phase arrest compared to GEM monotherapy, while the opposite was observed when GEM was combined with a high QUE concentration (200 μM). The combined treatment increases the apoptosis rate, upregulates p53 and cleaves caspase-3. The combination of GEM with a high concentration of QUE (200 μM) upregulated the MDR1 gene, which decreased the intracellular drug concentration and caused drug resistance.

SOR is the gold-standard in the treatment of advanced HCC. Abdu et. al. investigated the effects of SOR and QUE alone, and in combination in the treatment of HCC, in vitro and in vivo [136]. The in vivo study was performed on a chemically induced HCC rat model. The combined treatment was more effective in suppressing nuclear Ki-67 overexpression, compared to treatment with SOR or QUE alone. It was more effective in restoring inflammation and oxidation markers and improved lipid profile compared to the treatment with SOR alone. QUE used alone or in combination with SOR reduced the levels of tumor biomarkers (PIVKA-II and AFP), much more effectively than SOR alone. The combined treatment was more effective in inhibiting cancer progression, and the growth of hepatic tumor nodules and it restored the structural integrity of the liver. QUE alone or combined with SOR downregulated angiogenesis-related genes: TNF-α, VEGF, P53 and NF-κB. The in vitro study was performed on HepG2 cell lines. The IC_50_ values were 107.7 μM for QUE, 10.9 μM for SOR, and for an equimolar mix of SOR + QUE 9.98 μM, with the estimated CI value of 0.54 indicating a synergistic effect. Both QUE and SOR + QUE exerted an anti-proliferative effect on HepG2 cells through the induction of apoptosis. SOR arrested the cell cycle at the S phase, QUE arrested the cell cycle in G1 and S phases, and SOR + QUE increased the cell population in the S phase. SOR induced early apoptosis (6%), and late apoptosis (12%), while QUE induced only late apoptosis (11%). The combination increased late apoptosis (15%), indicating a synergistic effect (Table 2, Figure 2).

#### 5.1.3. Gastric Cancer

QUE combined with DOX decreased the proliferation of KATO III gastric cells [137]. The IC_50_ values of QUE and DOX in KATO III cells were 50.37 μM and 0.87 μM, respectively. After combination with QUE, the IC_50_ value of DOX was 0.64 μM. The combined QUE + DOX treatment promotes DNA damage. QUE and DOX increased the expression levels of γH2AX which played a substantial role in the DNA damage response, while QUE + DOX combined treatment increased γH2AX levels even more drastically. The QUE + DOX combination increased the ROS levels. Additionally, it decreased cellular antioxidant defense, and levels of SOD, catalase, GPx, GR, and GSH S-transferase. As a result of ROS overproduction and antioxidant defense inhibition, QUE enhanced the chemotherapeutic effect of DOX. 

Hyunh et al. examined the effects of different flavones against CYR61-overexpressing human gastric adenocarcinoma AGS (AGS-cyr61) cells, which show remarkable resistance to 5-FU and adriamycin (ADR) [138]. Among the tested flavones, QUE had the lowest IC_50_ = 46 μM and reduced the viability of AGS-cyr61 cells compared with AGS cells. QUE treatment decreased CYR61, MRP1, and, NF-κB p65 levels and induced PARP cleavage in AGS-cyr61 cells. There are limited reports on agents that can target CYR61 signaling. Additionally, QUE inhibited colony formation and induced caspase-dependent apoptosis. After treatment with QUE, AGS-cyr61 cells showed morphological changes, including condensed chromatin and apoptotic bodies. QUE induced a dose-dependent increase in the sub-G1 population. Moreover, caspase-9, -7, and -3 levels were reduced and the levels of cleaved caspase-9, -7, -3 and PARP were increased. QUE inhibited colony formation of AGS-cyr61, while no inhibition was detected in AGS cells. QUE reversed drug resistance through the induction of apoptosis and the inhibition of colony formation in AGS-cyr61 cells. QUE suppressed migration and downregulated EMT-related proteins in AGS-cyr61. AGS-cyr61 cells treated with a combination of QUE and 5-FU or ADR in the sub-lethal range showed strong synergy with CI being 0.21–0.54 for 5-FU and CI being 0.18–0.34 for ADR.

IRI, prodrug and its metabolite, SN-38, are the first line chemotherapeutics for gastric cancer, and potent inhibitors of DNA topoisomerase I (Topo I). Lei et al. investigated the effect of QUE combined with IRI/SN-38 in the AGS human gastric cancer cell line in vitro and in vivo [139]. The in vitro study evaluated the efficacy of high-dose SN-38 compared to the combination of QUE with low-dose SN-38. Results showed that cell viability and the percentage of apoptosis in combined treatments with QUE and SN-38 were comparable to treatment with high-dose SN-38 alone. AGS cells treated with a high dose of SN-38 exhibited an upregulation of β-catenin expression. QUE alone or in combination with low-dose SN-38 exhibited lower levels of β-catenin. The in vivo study performed on the AGS xenograft mouse model showed that the combination of QUE and IRI modulated angiogenesis-associated and EMT-related factors, and inhibited metastasis-related factors. VEGF-A levels decreased in tumor tissues and plasma samples in the Q + IRI group, while there was no significant difference between the QUE and control groups. The tumor size was the smallest in the QUE + IRI group. QUE decreased COX-2 gene expression. EMT-related proteins, such as Twist1 and ITGβ6, were lower in combined treatments with QUE and low-dose IRI than in high-dose IRI alone (Table 2, Figure 2). 

#### 5.1.4. Breast Cancer

Liu et al. investigated the effect of QUE on the antitumor activity of CIS and its side effect, renal toxicity, in breast tumor-bearing mouse models [140]. Experimental groups were divided into four groups: control, QUE, CIS, and CIS + QUE. QUE acts synergistically with CIS on tumor growth. The tumor volume of the CIS + QUE group was significantly lower (54% decreased) compared to the CIS group (29% decreased). Renal γ-glutamyltranspeptidase and alkaline phosphatase activities were increased and the content of renal thiobarbituric acid reactive substance was decreased in the CIS + QUE group compared to the CIS group. Additionally, QUE decreased the CIS-induced renal toxicity, and the oxidative damage of renal tissue, as was evidenced by the decreased serum blood urea nitrogen and creatinine levels, and increased GGT and AP.

Docetaxel (DTX) is used in the case of metastatic breast cancer, but prolonged use leads to drug resistance and toxicity. Safi et al. investigated the effect of the combined treatment with DTX and QUE on the MDA-MB-231 human breast cancer cell line [141]. The IC_50_ values for DTX and QUE were 33 nM and 125 μM, respectively; DTX (7 nM) + QUE (95 μM) showed the greatest synergistic effects with CI 0.76. The IC_50_ dose of DTX is reduced seven-fold when combined with QUE. Combined treatment increased apoptosis by upregulating the p53 tumor suppressor gene, and BAX protein, while BCL2, AKT, ERK1/2, and STAT3 proteins were downregulated. DTX resistance is related to the activation of PI3K/AKT, MAPK/ERK, and JAK/STAT3 signaling pathways. QUE down-regulated the expression of AKT, which inhibits the pro-apoptotic factors such as BCL2-associated agonist of cell death (BAD) and forkhead box protein O (FOXO). The combined treatment synergistically suppresses ERK1/2, which activates BCL2, BIM, BMF, and PUMA. QUE alone or in combination with DTX reduces STAT3 protein. Altogether, QUE increases the sensitivity of MDA-MB-231 cells to DTX by inducing apoptosis and reducing cell survival. 

DOX is a first-line drug for breast cancer chemotherapy, but its cardiotoxicity limits the maximum dose. Li et al. investigated the effect of combined treatment of DOX and QUE in breast cancer MCF-7 and MDAMB-231 cells, non-tumoral MCF-10A mammary cells, and myocardial AC16 cells [142]. The combined treatment with a low dose of QUE increased the accumulation of DOX in breast cancer MCF-7 and MDAMB-231 cells, by downregulating the expression of efflux ABC transporters including P-gp, BCRP, and MRP1. However, combined treatment had an insignificant effect on the expressions of P-gp, BCRP, and MRP1 in non-tumoral mammary cells and myocardial cells, and the cytotoxicity of DOX on normal mammary cells and myocardial cells was slightly reduced. Altogether, the combination of DOX and QUE allows the use of lower doses of DOX, as it attenuates the toxic side effects of DOX on non-tumor cells. DOX exposure increases the contractile responses and attenuates relaxation to both endothelium-dependent and endothelium-independent vasodilators [39]. The combination of QUE with DOX decreased the contractile responses of aortic smooth muscles compared to DOX alone. DOX-induced vascular dysfunction occurs within one hour of aortic ring exposure to DOX. This is related to the effects of DOX on Ca^2+^ channels, the elevated intracellular Ca^2+^ concentration can lead to excessive ROS generation. QUE decreases the ROS concentration. On the other side, a high dose of QUE (98 μM/38 μM) decreased the chemotherapeutic effect of DOX on MCF-7 and MDA-MB-231 cell lines, with CI values of 3.2 and 2.0, respectively, indicating a strong antagonistic interaction with DOX. In the ductal carcinoma cell line T47D, the combined treatment exerted an additive effect. The strong antagonistic interaction between QUE and DOX in different breast cancer cell lines might be attributed to the strong antioxidant activity of QUE, which decreases the generation of DOX-related ROS. Antagonism in some breast cancer cells might be attributed to its strong estrogenic activity and its proliferative impact on estrogen receptor-positive breast cancer cells. QUE induced the intracellular accumulation of DOX, by downregulating P-gp, in T47D at a lower concentration compared to MDA-MB-231 cells, and showed no effect in MCF-7 cells, which could explain the additive effect on T47D cells. Additionally, the combined treatment showed a lower percentage of apoptotic cells compared to DOX used alone. QUE induced cell accumulation in the S phase and the G2/M phase within both MCF-7 and MDA-MB-231 cell lines. Despite the potent vascular protective effect of QUE against DOX-induced vascular toxicity, it might seriously attenuate its anticancer potencies. 

DOX combined with a cyclophosphamide (CP), AC regimen, is the most used therapy for TNBC chemotherapy, but also in this case the cardiotoxicity limits its use [143]. Cardiotoxicity is the result of oxidative stress and the inhibition of the ERK1/2 signaling path. QUE enhances the effect of DOX-CP treatment by inhibiting ERK1/2 in AC-treated TNBC cells, downregulates the expression of c-Myc, upregulates the expression of cleaved caspase-3, and inhibits the expression of MMP-9 that mediates cell migration. A low-dose of QUE exerts cardioprotective effects by enhancing the activity of ERK1/2 in myocardial cells, upregulating the expression of c-Myc that promotes cell proliferation, and downregulating the expression of cleaved caspase-3 that mediates cell death. High-dose QUE exerts cardiotoxic effects by inhibiting the activity of ERK1/2.

QUE synergistically increases the effect of 5-FU on growth inhibition and apoptosis of the MCF-7 breast cancer cell line [144]. The growth inhibition rate of 100 μM 5-FU in MCF-7 was 2%, whereas it reached 71%, following treatment with 100 μM 5-FU + 446 μM QUE. The best synergistic effect was obtained for a combination of 100 μM 5-FU and 446 μM QUE. The combined treatment allows up to a 3.3-fold reduction in 5-FU dose. QUE increased the apoptotic effect of 5-FU through increased caspase-9 activity and Bax, and p53 gene expression, and decreased Bcl2 gene expression. The combined treatment also decreased colony formation. The ability of breast cancer cells to metastasize to other tissues increases mortality. Roshanazadeh et al. studied the effects of 5-FU and QUE combination on MDA-MB-231 breast cancer cells and MRC5 human normal lung fibroblast cells [145]; QUE showed a highly selective inhibitory effect on tumor cells (3.39), compared to 5-FU (0.65). Both QUE and 5-FU used alone reduces the proliferation of cancer cells. QUE synergistically enhances the inhibitory effect of 5-FU on the proliferation of breast cancer cells. The lowest CI value (0.33) was obtained for the combination of 50 μM QUE and 25 μM 5-FU. QUE allows a reduction in the dose of 5-FU. Additionally, they examined the effects of the combination (50 μM of QUE and 25 μM of 5-FU) on the growth of MRC5 human normal lung fibroblast cells, and results showed that this combination did not reduce normal cell viability compared with each drug alone. The combined treatment reduced the rate of BC cell migration by 62% and the expression of gelatinase enzymes MMP-9 and MMP-2 genes, which have a key role in the metastasis of breast cancer cells. 5-FU reduced the expression of MMP-2/-9 genes by 0.85 and 0.8-fold, respectively, QUE by 0.8 and 0.77-fold, respectively, and the combination by 0.48 and 0.35-fold. 

Ozkan et al. investigated the efficacy of the combination of lonidamine (LND) with QUE on human MCF-7 breast cancer cells [146]. The results showed that the combined use of LND and QUE increased cytotoxicity compared to administration alone. The combination of QUE (80 and 100 μM) and LND (5 and 10 μM) decreased the cell proliferation rate and showed a stronger antiproliferative effect on the cells compared to treatment with LND or QUE alone. LND induced a cell cycle arrest in the G2/M phase, while QUE and LND + QUE arrested the cell cycle at the S point, indicating a synergistic effect. The combination induced apoptosis, increased caspase levels, and decreased MMP-2/-9 mRNA more potently than LND or QUE alone (Table 2, Figure 2). 

There were no clinical trials regarding the combined treatment of QUE and anticancer drugs, in the last five years. 

## 6. Resveratrol

RES is natural stilbene, a non-flavonoid polyphenol, synthesized in plants as a phytoalexin to protect them from pathogens and other environmental stresses. Natural sources of RES are red grapes (especially the grape skin), berries, peanuts, pistachios, and cocoa, but it can also be found in food products such as red wine, berry juices, jams, and chocolate [147,148]. RES exists in two isomeric forms, *cis-* and *trans-*, with *trans-* isomer being more abundant and more stable, with plentiful biological activities connected to its therapeutic properties [148,149]. RES is poorly soluble in water (<0.05 mg/mL) and although it has a high rate of absorption of about 70% after oral administration (25 mg dose), the bioavailability is low due to rapid metabolism in the liver and intestine [42,148,150]. Despite these limitations, numerous in vivo studies reported a multitude of RES biological effects that might be related to its affinity to human serum albumin and lipoproteins, which facilitate the entry of RES into different tissues [42]. Some pharmacological effects attributed to RES are cardioprotective, neuroprotective, anticancer, antidiabetic, anti-obesity, anti-aging, and antimicrobial [31,42,147,148,149,150,151,152]. Indeed, a vast number of studies have demonstrated that RES possesses antioxidant and anti-inflammatory properties and is able to affect gene expression and interfere with numerous signaling pathways [31,148,149,152,153,154], although these features could lead to some toxic effects of RES as well [42]. Considering the anticancer effect, special attention is paid to the chemo-sensitizing effect of RES on cancer cells that acquired resistance towards chemotherapeutics [155,156]. MDR is a major obstacle and RES adjuvant therapy offers a possibility to circumvent this issue and ultimately have more success in contemporary cancer treatment.

### 6.1. Resveratrol Combined with Chemotherapy in Preclinical Studies

#### 6.1.1. Lung Cancer

**Figure 3:**
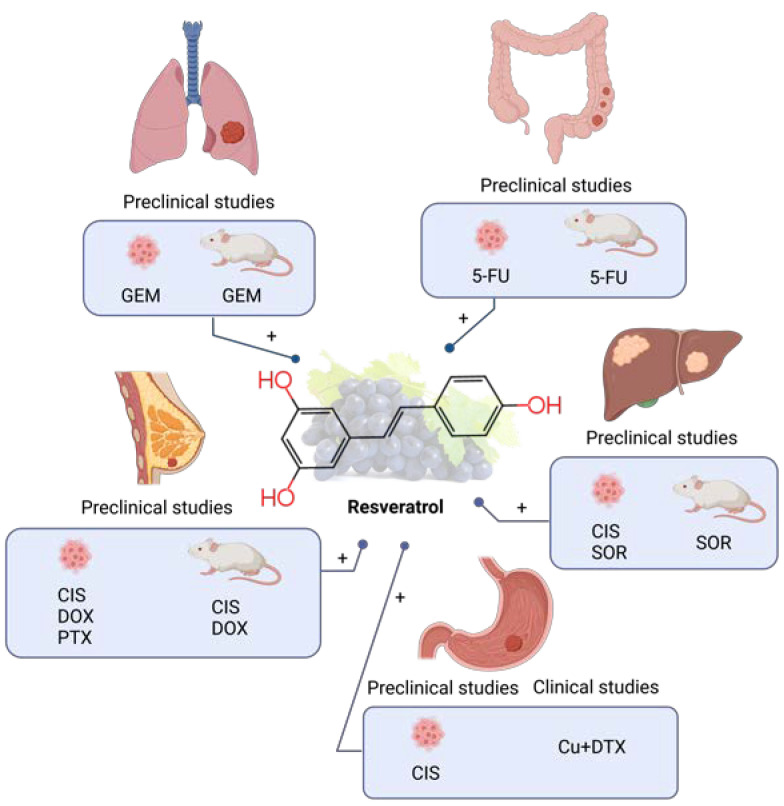
Combination of resveratrol and chemotherapy in preclinical (lung [157], colorectal 127-129], liver [104,158,159], gastric [151] and breast [65,160,161,162,163,164,165,166,167] cancer) and clinical (gastric cancer [168]) studies in the past five years. GEM: gemcitabine; 5-FU: 5-fluorouracil; CIS: cisplatin; SOR: sorafenib; DOX: doxorubicin; PTX: paclitaxel. Created with BioRender.com.

RES was investigated in combination with chemotherapeutic GEM in lung cancer HCC827 cells in vitro and in vivo [157]. RES was applied in 10 µM concentration together with 1 µM GEM in vitro but no synergistic effect on cell viability was observed (compared to GEM alone). Contrary, in vivo investigation showed that the administration of 25 mg kg^−1^ of GEM i.p. twice weekly and 1 µmol kg^−1^ RES five times weekly reduced the growth rate, weight, and volume of the tumor after 25 days of administration. This positive observation was attributed to the effect of RES to promote the tumor microvessel growth and blood perfusion of the tumor in HCC827 xenograft-bearing nude mice. The effect of RES on angiogenesis was further investigated and explained. RES downregulated both mRNA and protein levels of endoglin (ENG), a crucial protein in angiogenesis, in HCC827 cells in vitro, but also in vivo as was demonstrated by endoglin-positive staining of tumor tissue sections. The possible explanation of RES-induced tumor microvessel growth was given in the HCC827-HUVEC co-culture model where the activation of the ERK signaling pathway was observed and connected with a lower ENG expression level (Table 3, Figure 3). 

#### 6.1.2. Colorectal Cancer

The in vivo study on male albino rats showed the beneficial effect of RES in combination with chemotherapeutic 5-FU in N-methylnitrosourea-induced colon cancer [169]. The rats in the colon cancer group were treated with RES 10 mg/kg b.w. orally on daily basis and with 5-FU i.p. injected in doses of 12.5 mg/kg b.w. on days 1, 3, and 5 with the cycle being repeated every 4 weeks for 4 months. While the sole 5-FU treatment in N-methylnitrosourea-treated rats showed cytotoxicity through the activation of NF-κB and a significant increase in COX-2 level, the combination with RES effectively decreased NF-κB along with a reduction in COX-2. Additionally, in presence of RES, the p53 gene expression in colon tissue was induced to a value that corresponds to those in the control group of healthy rats. A combination of RES and 5-FU was also studied in vitro on DLD1 and HCT116 colon cancer cells and the results showed that RES can re-sensitize cancer cells to 5-FU chemotherapy [170]. The addition of RES to 5-FU led to enhanced cytotoxicity, induced S-phase cell cycle arrest and enhanced apoptosis of colon cancer cells. Results suggest these anti-proliferative effects were a consequence of pAkt inhibition. Furthermore, the pSTAT3 inhibition and decreased telomerase activity were in line with CD44 CSC biomarker abolition observed after RES and 5-FU combination treatment. Another study investigated the effect of RES and 5-FU co-treatment in HCT116 and HCT116R (5-FU-chemoresistant clone cells) in a TNF-β-mediated inflammatory tumor microenvironment in monolayer and 3D-alginate culture model [171]. The results showed dramatically enhanced inhibition of the invasion ability of HCT116 and HCT116R cells and downregulation of colon CSC markers ALDH1, CD44 and CD133 due to the presence of RES, compared to control in a 3D alginate culture. Moreover, RES and 5-FU co-treatment of HCT116 and HCT116R cells significantly induced caspase-dependent apoptosis regardless of a pro-inflammatory environment caused by TNF-β, indicating that RES may sensitize chemo-resistant HCT116R cells. RES efficiently suppressed the expression of NF-κB, MMP-9 and CXCR4 involved in invasion and metastasis in both cell populations. Additionally, the combination of RES and 5-FU showed marked suppression of vimentin, the transcription factor slug and the induction of E-cadherin expression, all of them being engaged in EMT in colon cancer cells (Table 3, Figure 3).

#### 6.1.3. Liver Cancer

The synergistic effect of RES and CIS was investigated in HCC C3A and SMCC7721 cells [158]. RES was shown to enhance the apoptosis induced by CIS and to significantly reduce the glutamine transporter ASCT2 expression and glutamine uptake in C3A and SMCC7721 cells. In that way, the conversion of glutamine to glutathione, the primary ROS-scavenging system in the cell, is obstructed. Furthermore, RES and CIS co-treatment markedly increased ROS production and DNA damage, which may be a consequence of affecting the expression of mitochondrial and cytoplasmic cytochrome c, caspase-9 and activated caspase-3. 

Another chemotherapeutic agent investigated in combination with RES in HCC HepG2 and Huh7 cell lines and in BALB/c mice xenografts was SOR [159]. Co-treatment of RES and SOR in vitro showed significant synergistic antiproliferative effects in comparison with the corresponding RES and SOR treatments alone in both cell lines. Accumulation of cells in S phase and a decrease in G0/G1 phase was observed. Cell cycle arrest in S phase was at least partly associated with decreased levels of CDK2 and CDC25A regulatory proteins and increased levels of cyclin A. The percentage of apoptotic cells was significantly increased in the co-treatment group compared with RES and SOR treatments alone, associated with increased levels of cleaved caspase-3, caspase-8, and caspase-9 proteins. In addition, the expression of PKA, p-AMPK, and eEF2K was decreased in HCC cells, suggesting that PKA/AMPK/eEF2K signaling pathways may be involved in the synergistic effect of RES and SOR when applied together. The in vivo investigation showed a significant reduction in relative tumor volumes and tumor weights in BALB/c mice xenografts in the case of RES and SOR co-treatment when compared with treatments alone. A study conducted by Bahman et al. also investigated the effect of a combination treatment of RES and SOR in human HCC Hep3b and HepG2 cells in different administration schedules and RES (as well as some other polyphenols) potentiated the lethality of SOR in a dose-, cell type- and administration schedule-dependent manner [104] (Table 3, Figure 3).

#### 6.1.4. Gastric Cancer

RES was investigated in combination with CIS in human gastric cancer AGS cells [151]. The results showed the viability was significantly suppressed and the apoptotic rate of AGS cells was increased when compared with treatments of RES or CIS alone. The molecular mechanism underlying the proapoptotic effect of RES and CIS co-treatment was further investigated. Proapoptotic protein Bax and the cleaved form of PARP were upregulated whereas the expression of the antiapoptotic protein Bcl-2 was downregulated relative to the CIS treatment alone. Moreover, increased PERK, p-eIF2α and CHOP protein levels revealed that the PERK/eIF2α/ATF4/CHOP signaling pathway, an important modulator of ER stress-mediated apoptosis, was activated by RES and CIS co-treatment. Indeed, a great increase in cytosolic Ca^2+^ levels found in this study after the administration of RES and CIS were in line with the activation of the ER stress-mediated apoptotic signaling pathway. In addition, RES and CIS co-treatment significantly induced G2/M cell cycle arrest by the upregulation of the proteins p-CDK1 (Tyr15), p21^Waf1/Cip1^ and p27^Kip^, the downregulation of Cdc25C expression, and the reduction in cyclin B1 protein expression (Table 3, Figure 3). 

#### 6.1.5. Breast Cancer

An extensive study of the synergistic effects of RES combined with CIS on a TNBC model (MDA-MB-231 cells) in vitro and in vivo was performed recently [160]. RES and CIS co-treatment showed a significant decrease in cell viability in a dose- and time-dependent manner, and this effect was synergistic. It was concluded that high doses of RES (185 μM) could enhance the efficacy of low doses of CIS in inhibiting tumor cell growth. Moreover, migration and invasion of MDA-MB-231 cells were significantly inhibited after the RES and CIS co-treatment. To investigate the underlying mechanism of this inhibition effect, MDA-MB-231 cells were treated with TGF-β1 to induce the changes in the expression of epithelial and mesenchymal molecular markers E-cadherin, vimentin, and fibronectin. RES and CIS co-treatment of TGF-β1-treated cells could reverse the effect of TGF-β1 on EMT markers. The results indicated that RES and CIS could be involved in the regulation of PI3K/AKT and Smad, and related to the regulation of NF-κB, JNK, and ERK. This observation was confirmed in vivo by analysis of the protein expressions in tumor tissues of MDA-MB-231 xenografts. RES could enhance the anti-tumor effect of CIS and significantly reduce the tumor weight in vivo, as well as alleviate the side effects of CIS. The effect of RES and CIS co-treatment on MDA-MB-231 breast cancer cells was investigated also by Özdemïr et al. [161]. The results of this study showed a significantly higher percent of apoptosis in cases when RES and CIS were applied together which allows for lower doses of CIS to be used in treatment. The activation of the caspase-9 and caspase-3 enzymes important in apoptosis was increased with maximal values achieved after the combination treatment of RES and CIS in MDA-MB-231 cells. These observations were accompanied by a higher percentage of mitochondrial membrane depolarization in co-treated cells which is often an indicator of early apoptosis. Another study of RES and CIS combination treatment was conducted on MCF-7 and T47-D (both estrogen receptor-positive cells) and MDA-MB-231 (estrogen receptor-negative cells) [162]. The antiproliferative effect of CIS was significantly enhanced after the addition of RES (50 µM and 100 µM) in all breast cancer cell lines. When MCF-7 cells were treated with 2 µM of CIS and 100 µM of RES the IC_50_ value for CIS decreased dramatically and this effect could be due to the ability of 100 μM RES to reduce the homologous recombination (HR) initiation complex mRNA components. Further investigation of RES and CIS co-treatment revealed a reduction in HR activity (due to decreased Rad51, Nbs-1, Mre-11 and Rad50) by RES that could explain the suppressed repair of DNA damage caused by CIS in MCF-7 cells. RES and CIS were applied also to MCF-7R cells (resistant to CIS), and results showed that RES might re-sensitize cells to CIS. Similarly, as in MCF-7 cells, RES was able to suppress Rad51 and at least partially inhibit the repair of DNA damage in MCF-7R cells. 

Several studies investigated the combination treatment of RES and DOX in breast cancer cells in vitro and in vivo [65,163,164]. Vargas et al. studied the effect of 30 µM RES and 100 nM DOX in MCF-7 cells and found a synergistic effect (CI 0.8) [163]. Cell viability was determined after 24 h and cells were replated in a drug-free medium for 15 days to assess the long-term effects of RES. Results showed that RES potentiated the long-term toxicity of DOX, and this effect may be due to the long-term increase in apoptosis and senescence by RES in DOX-treated MCF-7 cells. The effect of RES and DOX in MCF-7/ADR (DOX resistant cells) was investigated to examine how RES could sensitize breast cancer cells to DOX therapy [164]. RES and DOX co-treatment showed the strongest inhibitory effect on MCF-7/ADR cells and colony formation relative to the RES or DOX treatment alone. Moreover, combination treatment induced cell apoptosis and a stronger migration-inhibitory effect than treatment by RES or DOX alone. The study conducted by Zhang et al. investigated the effect of RES and Adriamycin (trade name for DOX, 2 mg/mL solution) on MCF-7-ADR cells with a special focus on miRNA modulation [65]. RES and DOX co-treatment in MCF-7-ADR cells and MCF-7 cells as control, significantly decreased drug tolerance and the IC_50_ value of DOX in a dose-dependent manner. The activation of caspase-8 and caspase-9, inhibition of proliferation and decreased cell viability in drug-resistant MCF-7-ADR were observed and attributed to the synergistic effect of RES and DOX. Apoptosis related miRNA miR-122-5p was upregulated and miR-542-3p downregulated in MCF-7-ADR cells treated with RES and DOX and the expression levels of targeted proteins of these miRNAs, Bcl-2, CDK2, CDK4, and CDK6, were significantly reduced. Further investigation in MCF-7-ADR cells transfected with miR-122-5p inhibitor confirmed that miR-122-5p is a key miRNA in RES re-sensitization of DOX-resistant breast cancer cells. Chen and co-workers also studied the effect of RES and DOX combination treatment in MCF-7/DOX cells (DOX-resistant MCF-7 cells) which resulted in notable inhibition of the growth activity and propagation ability of cells [165]. Apoptosis was synergistically induced, and the migratory ability of cells was strongly inhibited in the case of RES and DOX co-treatment in MCF-7/DOX cells. Taken together, RES was able to restore the DOX-sensibility of MCF-7/DOX in vitro. In vivo, RES and DOX could synergistically reduce the tumor volume and immunohistochemical staining showed a significant increase in the expression of PI3K and cleaved caspase-3 and markedly reduced p70 S6K and Ki67 expression in MCF-7/DOX xenografts in nude mice. Another in vivo study investigated the effect of RES and DOX on the absorption of 99mTc-MIBI in MCF-7 xenografts in mice [166]. 99mTc-MIBI uptake in MCF-7 cells was significantly reduced in the group of mice that received RES and DOX therapy, due to higher apoptosis in tumor cells. In addition, the severity of pathological injuries on the liver and heart cells was reduced after RES and DOX treatment, compared to the DOX group. 

Cipolletti et al. studied the effect of RES and PTX on MCF-7 breast cancer cells and showed that the pretreatment of cells with 1 μM RES could significantly increase the effect of 100 nM PTX on apoptosis via PARP-1 cleavage while the co-treatment of RES and PTX had not shown significant effect [167]. As was demonstrated in this study, RES could act as an ERα antagonist and decrease NGB levels due to impairment of the E2/ERα/NGB pathway. RES-enhanced sensitivity of MCF-7 cells to PTX could allow the use of lower doses of this chemotherapeutic in the clinical treatment of breast malignancies (Table 3, Figure 3).

### 6.2. Clinical Studies of Resveratrol Combined with Chemotherapy

The combination of RES and copper (Cu) was investigated in a clinical trial (CTRI/2019/07/020289) in patients with advanced gastric cancer that received docetaxel-based triplet chemotherapy [168]. The aim of that single armed phase II study was to assess the efficacy of RES and Cu in ameliorating the hematological and non-hematological toxic side effects of applied chemotherapy. Previous pre-clinical studies showed that chemotherapy toxicity is caused by cell-free chromatin particles (cfChPs) released from dying cells. These cfChPs could cause damage in healthy cells leading to inflammation, apoptosis, and more cell death, prolonging the toxic effects of chemotherapy. RES in presence of Cu could react as a pro-oxidant and deactivate cfChPs in vitro and in vivo. In this clinical trial, patients were receiving 5.6 mg of RES and 560 ng of Cu orally, three times a day, and docetaxel-based triplet chemotherapy every two weeks. The results showed that this combination therapy markedly reduced the incidence of non-hematological toxicities such as hand-foot syndrome, diarrhea, and vomiting, which could be helpful to retain the continuity of chemotherapy treatment and improve outcomes. Unfortunately, the addition of RES and Cu to docetaxel-based triplet chemotherapy could not reduce hematological grade ≥ 3 toxicity such as neutropenia or febrile neutropenia and the overall cumulative incidence of grade ≥ 3 toxicity. Altogether, this study represents a successful translation of preclinical findings to clinical practice, offering an improvement in the chemotherapy treatment of advanced gastric cancer (Figure 3).

## 7. Epigallocatechin Gallate

EGCG, a major green tea (*Camelia sinensis*) polyphenol constituent, has been the subject of extensive scientific research over the last few decades. EGCG is well known for a wide variety of biological properties, which include antioxidant, anti-inflammatory and anticancer effects, as well as neuro-, cardio- and vasoprotective actions [172]. Its chemopreventive and chemotherapeutic implications in cancer have been studied in detail. In lung cancer, EGCG has been described as a potent inducer of p53-dependent apoptosis and was also found to suppress cell invasion, hindering the cancer metastatic potential, by interacting with matrix metalloproteases. In colorectal cancer cells, EGCG exerted its antineoplastic pharmacological features by suppressing cancer cell proliferation, while an AMP-activated protein kinase-mediated apoptotic effect was observed in EGCG-treated colon cancer cells and human hepatoma cells [172,173]. Aside from a considerable number of promising in vitro results, EGCG has been shown to affect a plethora of different signaling pathways and transcription factors in vivo as well. EGCG successfully suppressed tumor growth by inhibiting tumor-associated macrophage infiltration and M2 macrophage polarization in a murine breast cancer model. It also inhibited IL-6-induced VEGF expression and angiogenesis in an AGS human gastric cancer cell xenograft model by suppressing Stat3 activity [174]. 

### 7.1. Epigallocatechin Gallate Combined with Chemotherapy in Preclinical Studies

#### 7.1.1. Lung Cancer

**Figure 4:**
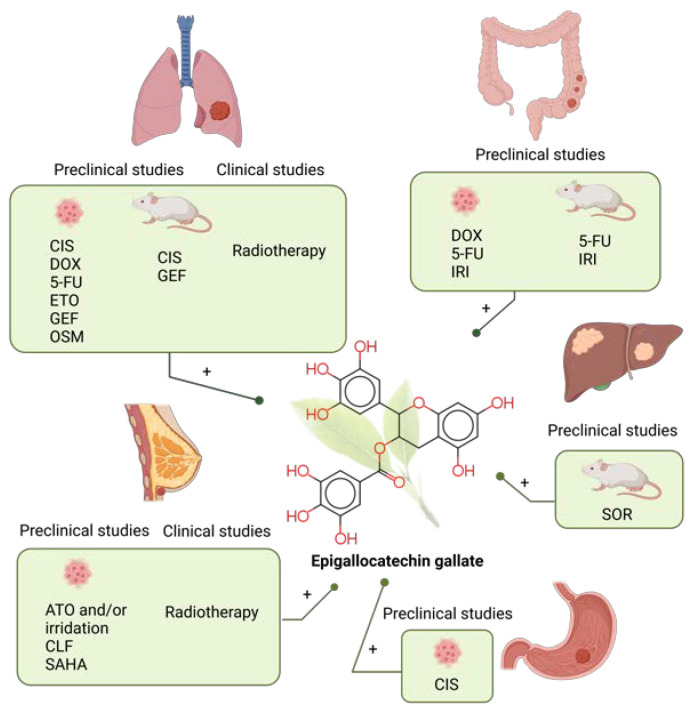
Combination of epigallocatechin gallate and chemotherapy in preclinical (lung [175,176,177,178,179,180], colorectal [181,182,183,184,185,186,187], liver [188], gastric [189] and breast [190,191,192,193] cancer) and clinical (lung [194,195,196] and breast [197,198] cancer) studies in the past five years.; CIS: cisplatin; DOX: doxorubicin; 5-FU: 5-fluorouracil; ETO: etoposide; GEF: gefitinib; OSM: osimertinib; IRI: irinotecan; SOR: sorafenib; ATO: arsenic trioxide; CLF: clofarabine; SAHA: suberoylanilide hydroxamic acid. Created with BioRender.com.

Considering all the exceptional anti-cancer properties of EGCG, an increasing number of studies are now focusing on determining the possible synergistic effects of EGCG and cancer chemotherapeutics. EGCG combination therapy in lung cancer has been comprehensively investigated. Deng et al. demonstrated that EGCG synergistically potentiated CIS antitumor efficacy in the A549 lung adenocarcinoma cancer cell xenograft model by increasing CIS concentration in tumor tissue, especially when CIS was applied during the vascular normalization window [175]. Furthermore, it was reported that EGCG enhanced sensitivity to CIS in a lung cancer cell line by targeting the DNA repair endonuclease ERCC1/XPF activity [176]. Moreover, using EGCG as an adjuvant has been shown to affect drug uptake and retention. It has been observed that EGCG restored DOX responsiveness by decreasing drug efflux and MDR signaling and invasiveness, while at the same time increasing drug internalization and participating in the modulation of redox signaling [177]. Similarly, EGCG sensitized lung adenocarcinoma cells towards etoposide (ETO) by maintaining Nrf2-mediated redox homeostasis and by increasing intracellular uptake and retention of ETO, which, in turn, augmented cell death [178]. Polonio-Alcalá et al. demonstrated that EGCG combination with either GEF or osimertinib (OSM) resulted in mostly additive effects [179], while another study described how EGCG overcame GEF resistance in NSCLC through inhibiting autophagy and enhancing cell death by inhibiting the Raf/MEK/ERK pathway [180] (Table 4, Figure 4).

#### 7.1.2. Colorectal Cancer

EGCG combination with chemotherapy drugs has been thoroughly investigated in colorectal/colon cancer as well. It has been revealed that EGCG combined with either DOX or 5-FU successfully reduced cancer cell growth in HCT15 colon cancer and A549 lung cancer cell lines [181]. Moreover, EGCG effectively decreased the dose of DOX needed to reach cytotoxicity by mediating P-gp activity in the Caco-2 cell line [182]. Similarly, EGCG and 5-FU synergistic effects have been reported in other colon and CRC cell lines as well [183,184]. La et al. carried out an in vitro and in vivo investigation and described how EGCG managed to enhance the sensitivity of colon cancer cells to 5-FU by inhibiting the GRP78/NF-κB/miR-155-5p/MDR1 pathway and by promoting 5-FU accumulation in cancer cells [185]. Lastly, Wu et al. examined the combined anticancer effects of EGCG and IRI against RKO and HCT116 CRC cells and in HCT116 transplanted BALB/c nude mice xenografts. It was reported that EGCG increased the sensitivity of CRC cells to IRI through GRP78-mediated endoplasmic reticulum stress [186], enhanced DNA damage in cancer cells, induced cell apoptosis and prevented cancer cell migration and invasion [187] (Table 4, Figure 4).

#### 7.1.3. Liver Cancer

Certain studies have been conducted to better understand the possible synergistic actions of EGCG and different chemotherapeutic agents in liver cancer. Specifically, the co-administration of EGCG and SOR in a diethyl nitrosamine induced HCC model in Wistar albino rats resulted in enhanced chemoprotection and a comparable effect with a standard dose of SOR. The protective effects were evident in a satisfying decline in tissue degeneration and hyperchromatism, significantly lower liver enzyme levels and in a greater antioxidant capacity [188] (Table 4, Figure 4). 

#### 7.1.4. Gastric Cancer

EGCG and chemotherapy combination effects have also been investigated in gastric cancer. Namely, EGCG enhanced CIS anticancer effect against gastric cancer BGC-823 cells in vitro by modulating the p19^Arf^-p53-p21^Cip1^ signaling pathway. The combination therapy resulted in significant nuclear shrinkage and a reduction in the proliferation rate, cloning efficiency, and cancer cell migration [189] (Table 4, Figure 4). 

#### 7.1.5. Breast Cancer

Concomitant use of EGCG and cytostatic agents in breast cancer has also been shown to generate promising results. For instance, combination therapy which included EGCG and arsenic trioxide (ATO) with or without radiation, displayed synergistic effects visible in the rise of cancer cell death in the MCF-7 breast cancer cell line [190]. Likewise, it was demonstrated that a concurrent EGCG and clofarabine (CLF) use in vitro epigenetically affected *RARB* expression, synergistically inhibited breast cancer cell growth and successfully induced apoptosis [191]. Lastly, certain authors explored and emphasized the combinatorial effects of EGCG and suberoylanilide hydroxamic acid (SAHA) in multiple TNBC cell lines. Steed et al. highlighted that the proposed polyphenol and drug combination successfully induced cancer cell apoptosis, possibly through the epigenetic modulation of cIAP2 expression [192]. Similarly, Lewis et al. described changes in ERα, E- and N-cadherin, oncogenic miR-221/222 and tumor suppressors p27 and PTEN expression levels and the consequential successful regulation of cancer growth, proliferation, and migration, as a result of EGCG and SAHA co-administration [193] (Table 4, Figure 4).

### 7.2. Clinical Studies of Epigallocatechin Gallate Combined with Chemotherapy

Several clinical studies regarding concurrent EGCG and conventional cancer therapy use have been conducted over the past few years. Recent trials involving EGCG and radiotherapy have primarily been focused on investigating the possible ameliorating effects of EGCG on cancer treatment side effects and the proposed evidence suggests EGCG could exert certain radioprotective properties in lung and breast cancer patients undergoing radiotherapy cancer treatment [194,195,197].

Namely, in a prospective, three-arm, randomized and controlled phase 2 clinical study (NCT02577393), Zhao et al. investigated the potential benefits of EGCG administration in the prevention and treatment of acute radiation-induced esophagitis (ARIE) in lung cancer patients receiving radiotherapy. A total of 83 patients enrolled in the study were divided into three groups and given either a 440 μmol/L solution of EGCG orally three times a day, as a prevention or treatment, or conventional ARIE therapy. Statistically significant differences in the maximum esophagitis grade were detected among the three groups. Furthermore, the maximum ARIE observed in patients receiving EGCG was significantly lower than in conventionally treated patients. In addition, the mean adjusted esophagitis index (AEI) differed among groups, being the lowest in the EGCG prevention group and the highest in the conventional ARIE therapy group. Lastly, the adjusted pain index (API) and the adjusted dysphagia index were shown to be significantly lower in patients receiving EGCG than in patients receiving conventional ARIE therapy [194,196].

Subsequently, a 5-year observational, non-interventional survival analysis of the aforementioned phase 2 study (NCT02577393) was conducted. The analysis included 38 patients with small cell lung cancer (SCLC) who received EGCG or conventional ARIE therapy in the NCT02577393 study. Radiation response rate and PFS were assessed as primary endpoints of the study, while secondary endpoints included OS and EGCG efficacy in esophagitis treatment. The results indicated that the ORR in the EGCG group was higher than in the conventionally treated group, while median PFS and OS were not markedly extended. The 5-year PFS in the EGCG and conventional therapy group were 33% and 9.3%, respectively, while the 5-year OS was 30.3% and 33.3%, respectively. Additionally, ARIE symptom analysis indicated that the mean AEI and API in the EGCG group were lower than those of conventionally treated patients. Finally, the authors pointed out that while the study was conducted on a relatively small sample, the results suggest EGCG could bring a clinical benefit in SCLC patients undergoing radiotherapy cancer treatment, though further research is warranted [195] (Figure 4).

On a similar note, a double-blind, placebo-controlled, phase 2 randomized clinical trial (NCT02580279) was conducted in order to assess EGCG efficacy in preventing dermatitis occurrence in breast cancer patients receiving postoperative radiotherapy. The trial included 180 participants who were randomly assigned into two groups and given either a 660 μmol/L EGCG solution or 0.9% NaCl saline as a placebo. The solutions were applied topically by spraying the entire radiation field starting from the first day of radiation and ending 2 weeks after radiation completion. Incidence of grade 2 or worse radiation-induced dermatitis (RID) was assessed as the primary endpoint of the study, while secondary endpoints included RID index (RIDI), symptom index, changes in skin temperature and safety. The results indicated that grade 2 or worse RID occurrence, mean RIDI and symptom indexes were all significantly lower in patients receiving EGCG, compared to the placebo group. The authors concluded that topical EGCG use markedly reduced RID occurrence and intensity in breast cancer patients receiving postoperative radiotherapy [197,198] (Figure 4).

## 8. Apigenin

AP is a flavonoid found in various vegetables (parsley, celery, garlic), fruit (orange, grapefruit) and other plants (chamomile, thyme, oregano, mint, rosemary, sage) [199,200]. AP is usually stored in plants in a water-soluble glycosylated form. Purified AP is a yellow powder, almost insoluble in water and well soluble in organic solvents such as dimethylformamide, dimethylsulfoxide and ethanol [48,201]. 

AP has shown antioxidative, anti-inflammatory, antiproliferative and proapoptotic effects, and thus a beneficial effect in obesity, diabetes, hypertension, cardiovascular diseases, Parkinson’s and Alzheimer’s diseases [48,201,202]. Antitumor activity has been observed in various types of cancer (breast, colon, liver, lung, etc.). AP exerts its antitumor effects by regulating various signaling pathways and processes including apoptosis, angiogenesis, cell cycle, inflammation and tumor suppressor genes [202].

The poor solubility and low bioavailability of AP can be improved by different delivery systems such as nanoparticles, micelles, liposomes, and dendrimers [48].

Numerous studies have confirmed the ability of AP to increase the effectiveness and alleviate the side effects of classical chemotherapeutic drugs such as CIS, DOX, 5-FU, etc. [48,199]. The combination of AP and chemotherapy has shown anti-cancer effects such as a reduction in cell proliferation, stimulation of apoptosis, and metastases suppression [199].

### 8.1. Apigenin Combined with Chemotherapy in Preclinical Studies

#### 8.1.1. Lung Cancer

The influence of AP on the antitumor efficacy of CIS in the treatment of NSCLC whose stem cells develop resistance to CIS was examined. AP enhanced the antitumor effect and eliminated cancer stem cells in A549, H1299 and A549R cell lines [203]. By inhibiting histone deacetylase and modulating apoptotic and cell cycle regulatory genes, AP enhanced the anticancer effect of CIS on A549, H460 and H1299 cell lines [204].

**Figure 5:**
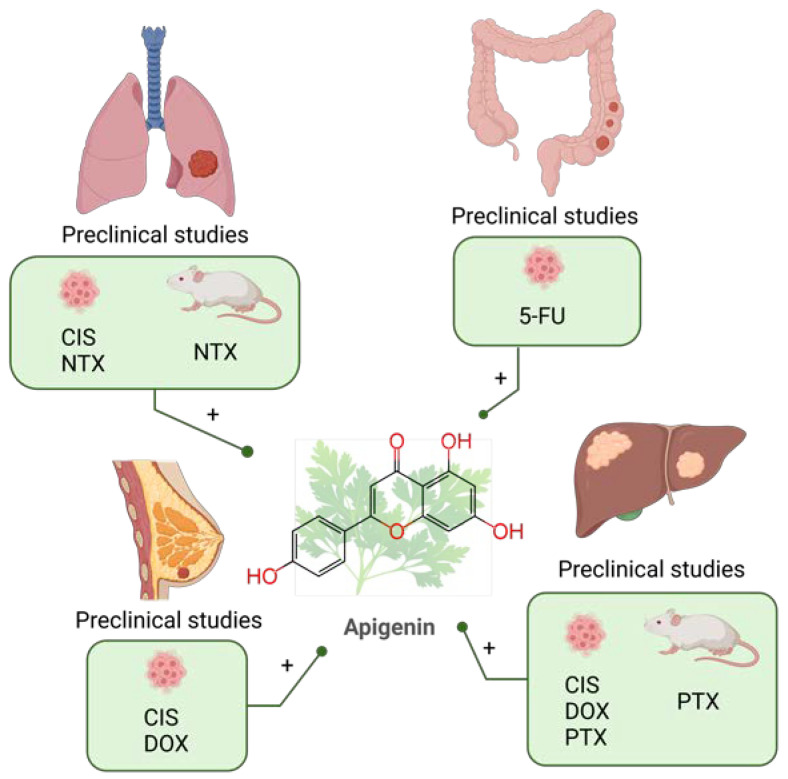
Combination of apigenin and chemotherapy in preclinical (lung [203,205], colorectal [184,206], liver [207,208,209] and breast [210,211] cancer) studies in the past five years. CIS: cisplatin; NTX: navitoclax; 5-FU: 5-fluorouracil; DOX: doxorubicin; PTX: paclitaxel. Created with BioRender.com.

AP has also been combined with the experimental anti-cancer drug ABT-263. AP increased the expression of Noxa in EGFRm tumor cells and thus acted synergistically with ABT-263 to suppress the growth and proliferation of tumor cells in vitro and in vivo [205] (Table 5, Figure 5).

**Table 5:** Table summarizing the combination therapy in the past five years of apigenin with chemotherapy in the preclinical studies in vitro (cell lines) and in vivo (rodents).

Cancer Type	Chemotherapy	Dosage	Assay Type	Molecular Effect	Study Conclusion	Ref.
Lung cancer	CIS	5–20 µM AP + 10 µM CIS for A549 cells; 10–20 µM AP + 10 µM CIS for A549R cells; 20–30 µM AP + 20 µM CIS for H1299 cells	A549, A549R and H1299 cell lines	Upregulation of p53.	AP enhanced the antitumor effect of CIS in A549, H1299, and A549R cells.	[203]
		AP:CIS drug ratio 5:1 for A549 cells; 10:1 for H460 cells; 4:1 for H1299 cells	A549, H460 and H1299 cell lines	S phase prolongation and G2/M cell cycle arrest. Inducing p21 and PUMA.	AP enhances the anticancer effect of CIS by inducing apoptosis and arresting the cell cycle.	[18]
	NTX	In vitro: 10–20 μM AP + 1–2 μM ABT-263. In vivo: 25 mg/kg AP + 100 mg/kg ABT-263	H1975, HCC827, H1650 and H3255 cell lines and BALB/c nude mice	Upregulated the expression of Noxa by targeting the AKT-FoxO3a pathway and inhibited ERK.	AP synergized with ABT-263 by suppressing the growth and proliferation of tumor cells in vitro and in vivo.	[205]
Colorectal cancer	5-FU	20 µM AP + 20 µM 5-FU	HCT116 and HT29 cell lines	Inhibited the upregulation of TS induced by 5-FU. Increasing reactive oxygen species production, intracellular and intramitochondrial Ca^2+^ concentrations, and mitochondrial membrane potential.	AP enhanced the efficacy of 5-FU by potentiating HCT116 cell apoptosis and enhancing cell cycle disruption. Acquired resistance to 5-FU was reduced.	[206]
		0.1–100 μM AP + 1 μM 5-FU	WiDr cell line		AP and 5-FU exerted synergistic effects in WiDr cells.	[184]
Liver cancer	CIS	10 μM–20 μM AP + 0.025–5 μg/mL CIS	HepG2, Hep3B, and Huh7 cell lines	Cell cycle delays during the first mitotic division in Hep3B and Huh7 cells and the second mitotic division in HepG2 cells.	AP enhanced the genotoxic, cytotoxic, anti-invasive and anti-migratory effects of CIS.	[207]
	DOX	25–200 μM AP + 1 μM DOX	HepG2 cell line	Inhibition of glycolytic genes expression (hexokinase 2 and lactate dehydrogenase A).	The combination of AP and DOX exhibited a very effective cytotoxic mechanism in HepG2 cells.	[208]
	PTX	In vitro: 40 mM AP + 6.25–100 nM PTX. In vivo: 1 mg/kg/day AP + 3.5 mg/kg/day PTX	HepG2 cell line and Balb/c nude mice	Suppressing the intratumoral expression of HIF-1a via inhibiting the AKT/p-AKT pathway and the expression of HSP90 simultaneously.	AP reduced hypoxia-induced PTX resistance in hypoxic tumors.	[209]
Breast cancer	CIS	5–100 μg/mL AP + 5–100 μg/mL CIS	MDA-MB-231 and HCC1806 cell lines	Inhibition of telomerase activity. Down-regulation of hTERT, Hsp90 and p23 at transcriptional and translational level.	AP and CIS synergistically inhibited telomerase activities by reducing the catalytic subunit of the enzyme.	[210]
	DOX	50 μM AP + 1 μM DOX	MCF-7 cell line	Reduced population of cells in G1 phase. Reduced AP site level. Increased phosphorylated H2AX in the nucleus.	AP enhanced the cytotoxic effect of DOX by increasing the formation of DNA damage and decreasing the expression of DNA repair genes.	[211]

AP: apigenin; CIS: cisplatin; NTX: navitoclax; 5-FU: 5-fluorouracil; DOX: doxorubicin; PTX: paclitaxel.

#### 8.1.2. Colorectal Cancer

The combination effect of AP with 5-FU was determined in CRC. AP suppressed the expression of thymidylate synthase and increased the sensitivity of HCT116 cells to 5-FU. Cell viability was inhibited and p53 expression was enhanced [206]. In another study, a synergistic effect was found on the WiDr colon cancer cell line [184] (Table 5, Figure 5).

#### 8.1.3. Liver Cancer

The ability of AP to improve the efficacy of chemotherapeutics was investigated in different liver cancer cell lines. Anti-invasive and anti-migratory activity was demonstrated by the combination of AP with CIS on HepG2, Hep3B, and Huh7 cell lines. While a synergistic genotoxic effect was observed on Hep3B cells, a less additive effect was observed on HepG2 and Huh7 cells [207]. Furthermore, the administration of AP with DOX significantly enhanced its cytotoxic effect on HepG2 cells by changing the expression of glycolytic pathway genes [208]. AP reduced hypoxia-induced PTX resistance in hypotoxic tumors as was demonstrated in vitro on HepG2 cells and in mouse models [209] (Table 5, Figure 5).

#### 8.1.4. Breast Cancer

The combination of AP with CIS was tested in TNBC and a synergistic effect was shown in MDA-MB-231 and HCC1806 cells with regard to telomerase activity inhibition [210]. Furthermore, AP enhanced the cytotoxic effect of DOX on MCF-7 cells by inhibiting the DNA damage response [211] (Table 5, Figure 5).

There are no available clinical studies of AP combined with chemotherapy in the last five years.

## 9. Conclusions

So far, the results of preclinical research on different cancer lines and on experimental animal models confirm the efficiency of combined chemotherapy with polyphenols, acting by different molecular mechanisms. Considering the large number of studies, curcumin could be a molecule of choice in future chemotherapy cocktails. Due to the existence of the ADMET process in vivo and the poor bioavailability of most polyphenols, it is necessary to carefully plan studies on humans. Some clinical studies dealing with combined therapy have been completed and some are still in progress. Despite promising results, the questions about optimal dose combinations, potential side effects and type of formulations remain open. Additionally, it should be kept in mind that some polyphenols can act antagonistically. Therefore, more studies are required to confirm and improve the efficiency of combined chemotherapy with polyphenols for oncology patients.

## Figures and Tables

**Table 1:** Table summarizing the combination therapy in the past five years of curcumin with chemotherapy in the preclinical studies in vitro (cell lines) and in vivo (rodents).

Cancer Type	Chemotherapy	Dosage	Assay Type	Molecular Effect	Study Conclusion	Ref.
Lung cancer	CIS	41 µM CUR + 30 µM CIS for A549 cells; 33 µM CUR + 7 µM CIS for H2170 cells	A549 and H2170 cell lines	Suppression of the self-renewal capability of cancer stem cells.	Synergistic inhibition of NSCLC.	[83]
		In vitro: 2–32 µM CUR + 0.5–8 µg/mL CIS. In vivo: 50 mg/kg CUR + 2.5 mg/kg CIS	A549, H1299, NCI-H460 cell lines and BALB/c mice	Upregulating the levels of CTR1 and Sp1 to increase more Pt^2+^ uptake.	Enhancing sensitivity and antitumor effects of CIS in NSCLC.	[85]
	CIS + X-ray	10 µmol/L CUR + 1 mg/L CIS	A549 cell line	Inhibition of EGFR-related signaling pathway.	Inhibition of cancer cell migration and invasiveness. Augmenting radio-sensitization effects against NSCLC.	[84]
	CZT	30 µM CUR + 20 µM CZT	A549, H460, H1299 and H1066 cell lines	Increasing the levels of miR-142-5p through epigenetic and suppressing autophagy.	Enhancing NSCLC sensitivity to CZT treatment.	[86]
	GEF	In vitro: 5–10 µM CUR + 0–20 µM GEF. In vivo: 1 g/kg CUR + 100 mg/kg GEF	H157, H1299, PC-9 cell lines and BALBL/c mice	Inhibition of Sp1/EGFR activity to induce autophagy mediated apoptosis.	Reduction in tumor volume. Elevating the sensitivity to GEF in NSCLC patients with mutated EGFR.	[87]
	GEM	3 µM CUR + 58.2 µM GEM for A549 cells; 3 µM CUR + 98.72 µM GEM for A549/GEM cells	A549 and A549/GEM drug-resistant cell lines	Downregulating expression of MMP9, vimentin, and *N*-cadherin and upregulating *E*-cadherin to slow EMT.	Elevating sensitivity of GEM-resistant NSCLC and decreasing migration and invasion.	[88]
	PTX	75, 25, 50% (*w*/*w*) CUR + 25, 50, 75% (*w*/*w*) PTX	Calu-3 and A549 cell lines	Induction of apoptosis/necrotic cell death and G2/M cell cycle arrests. Increased intracellular ROS, mitochondrial depolarization, and reduced ATP content.	The combination exerts a more potent cytotoxic effect. CUR neutralizes cytotoxic effects of PTX.	[89]
Colorectal cancer	CIS	10, 20 µM CUR + 0.4, 8 µM CIS	HT-29/CIS drug-resistant cell line	Inhibition glutamine through miR-137-mediated.	CUR treatment overcame CIS resistance and suppressed proliferation of CRC.	[90]
	5-FU	30 μM CUR + 20 mg/L 5-FU for HT-29 cells; 10 μM CUR + 10 mg/L 5-FU for SW480 cells	HT-29 and SW480 cell lines	G2/M Phase cell cycle arrest and downregulation of NNMT by p-STAT3 depression.	The combination inhibits CRC proliferation.	[91]
		In vitro and in vivo: 10 μM CUR + 5 μM 5-FU	SW620 cell line and female nude mice	Inhibited pERK signaling and downregulated L1 expression in SW620 cells.	Significantly increased apoptosis rate extended the survival of immunodeficient mice in the combination group as compared to that of the 5-FU group.	[92]
		5–40 μM CUR + 1.39 μg/mL 5-FU	rHCT-116/5-FU drug-resistant cell line	Regulating the TET1-NKD2-WNT signal pathway to inhibit the EMT progress.	CUR might exert an anti-resistant effect to 5-FU in HCT-116 cells.	[93]
		2–25 μg/mL CUR + 0.05–1000 μg/mL 5-FU	HCT-8 and HCT-8/5-FU drug-resistant cell lines	Downregulation of P-gp and HSP-27.	Inhibition of tumor growth. Reversal effects on MDR.	[94]
	IRI	In vitro: 2–14 μg/mL CUR + 2–14 ng/mL IRI. In vivo: 5 mg/kg CUR every other day 3 times + IRI 25 mg/kg every other day 3 times	CT-26 cell line and C57 BL/6j mice	Upregulated ICD-related proteins including CALR and HMGB1a.	CUR may synergistically improve the antitumor effect of IRI by promoting the immunogenic cell death (ICD) effect.	[95]
		100 mg/kg CUR by intragastric administration for 8 days + 75 mg/kg IRI for 4 days	IRI-treated BALB/c nude mice	Downregulation of NF-κB.	Protective effect against IRI-induced intestinal mucosal injury.	[96]
		2.5–20 µM CUR + 10–100 µM IRI	LoVo and LoVo/CPT-11 drug-resistant cell lines	Downregulation of CD44, EpCAM, CD24, Bcl-2 and upregulation of Bax.	Attenuated chemoresistance of CRC cells via targeting and inducing apoptosis in CRC.	[97]
		5–15 µg/mL CUR + 2.5–100 µg/mL IRI	LoVo/CPT-11R drug-resistant cell line	Increase of *E*-cadherin; downregulation of vimentin and *N*-cadherin.	Suppressed epithelial-mesenchymal transition (EMT).	[98]
	OXA	In vitro: HCT116 and SW480 cells 0–8 µM CUR + 0.5–32 µM OXA; HCT116/OXA cells 4 µM CUR + 8 µM OXA. In vivo: 60 mg/kg CUR + 10 mg/kg OXA	HCT116, SW480, HCT116/OXA drug-resistant cell lines and BALB/c nude mice	Inhibition of TGF-β/Smad2/Smad3 signaling.	Inhibition of cell proliferation and reduced tumor weight and volume.	[99]
	RG	15, 30 μM CUR + 0–1 μM RG	HCT-116 (KRAS mutant) and HT-29 (KRAS wild-type) cell lines	-	CUR behaved like MEK-specific inhibitor (U0126) to enhance RG-induced growth inhibition, apoptosis, and autophagy in HCT-116 cells.	[100]
Liver cancer	CXB	1.25–40 μM CUR + 3.125–100 μM CXB	HepG2 cell line	Inhibition of cell proliferation via the downregulation of NF-κβ, PGE2, MDA and Akt phosphorylation; suppression of cyclin D1 and VEGF expression. Increased apoptotic activities via the upregulation of caspase 3 activity.	Synergistic antiproliferative interaction. Possible use of lower and safer doses of CXB.	[38]
	CIS	15.6–500 μM CUR + 10, 25 μM CIS	HepG2 cell line	-	The combination of CIS with CUR inhibited cell viability and exhibited a significant agonist effect in selected cancer cells in a time and dose dependent manner.	[101]
	5-FU	In vitro: 5, 10 μM CUR + 2.5, 5, 10 μM 5-FU. In vivo: 56.65 mg/kg CUR + 10 mg/kg 5-FU	SMMC-7721, Bel-7402, HepG-2, MHCC97H cell lines and BALB/c nude mice	Decreased expression of NF-κB protein in the nucleus. Increased expression of NF-κB protein in cytoplasm. Downregulation of COX-2 expression.	Synergistic effects and in vivo tumor growth inhibition.	[102]
	PTX	5, 10, 20 μM CUR + 0.16–10.24 μM PTX	Hep3B and HepG2 cell lines	Downregulation of Lin28.	Synergistic effect. CUR increased the sensitivity of HCC cells to PTX.	[103]
	SOR	60, 120 μM CUR + 0.25–10 μM SOR	Hep3b and HepG2 cell lines	S-phase and G2/M phase arrest of liver cancer cells, induced apoptosis, reduced the protein levels of cyclins A, B2 and D1, phosphorylated retinoblastoma and B-cell lymphoma (Bcl), increased the protein levels of Bcl-2-associated X protein, cleaved caspase-3 and cleaved caspase-9.	CUR augmented the apoptosis-inducing potential of SOR.	[104]
Gastric cancer	DOX	2.5–30 μg/mL CUR + 2.5–30 μg/mL DOX	AGS cell line	Activation of pro-apoptotic protein Bax, repression of anti-apoptotic protein Bcl-2, upregulation of caspase 9 activity.	Decrease in AGS cell viability. Dose-dependent inhibition of cell invasion and migration.	[81]
Breast cancer	APA	25–100 μM CUR + 25–100 μM APA	MCF7 cell line	Induced apoptosis of breast cancer cells through increased expression of apoptosis-inducing BAX and SMAC genes as well as decreased expression of apoptosis inhibitor BCL2 and SURVIVIN genes.	Combination therapy exerts more profound anti-proliferation effects on breast cancer cells than APA or CUR monotherapy.	[105]
	CARB	5 µM CUR + 2 µM CARB	CAL-51, CAL-51-R and MDA-MB-231 cell lines	Increasing ROS production, which downregulated the DNA repair protein RAD51, leading to upregulation of γH2AX.	CUR sensitizes TNBC to the anticancer effect of CARB.	[106]
	DOX	25 µM CUR + 5 µM DOX	MCF-7/DOX drug-resistant cell line	Reduced the Aurora-A expression. Triggered P53 stabilization. Growth arrest and apoptosis induction.	Reversed DOX insensitivity and increased sensitivity in DOX-resistant MCF-7 and MCF-7 cell lines.	[107]
		10 µM CUR + 2.5–100 µM DOX	MCF-7/DOX and MDA-MB-231/DOX drug-resistant cell lines	Suppression of PI3K/Akt, GSK3β, β-catenin phosphorylation. Inhibition of efflux function of ABCB4 via the inhibition of ATPase activities of ABCB4. Inhibition of EMT via the upregulation of *E*-cadherin; downregulation of Smad2 phosphorylation.	Enhanced the sensitivity of breast cancer cells to DOX. Increased intracellular levels of DOX and reversed chemoresistance.	[108]
	LAP	1.5 μM CUR + 5, 20 nM LAP	AU-565 cell line	Inhibition of cell proliferation via the suppression of Akt phosphorylation. Reversal of HER2-induced chemoresistance via the downregulation of HER2.	Potentiated action of the metastasis treatment drug LAP.	[109]
	PTX	In vitro: 0.01, 0.1 µM CUR + 0.01–100 µM PAX. In vivo: 50 mg/kg CUR, p.o., 3 times/week + 10 mg/kg PTX, i.p., once/week	MCF-7EAC-tumor bearing mice	Inhibition of the ALDH-1 and PTX-induced Pgp-1 expression. Synergistic cytotoxic interaction via upregulation of Bax, caspase-7, -9 and downregulation of Bcl-2 expression. Inhibition of PTX-induced Pgp-1, and -ALDH-1 expression in the animal model.	Using CUR enhanced the tumor response to PTX.	[110]
		30 µM CUR + 10 nM PTX	MCF-7 and MDA-MB-234 cell lines	Increased caspase 3 activation, PARP cleavage, loss of membrane integrity. Increased apoptotic effect of PTX. Reduced PTX-induced NF-κB.	Higher level of apoptosis compared with either substance alone.	[111]

CUR: curcumin; CIS: cisplatin; CZT: crizotinib; GEF: gefitinib; GEM: gemcitabine; PTX: paclitaxel; 5-FU: 5-fluorouracil; IRI: irinotecan; OXA: oxaliplatin; RG: regorafenib; CXB: celecoxib; SOR: sorafenib; DOX: doxorubicin; APA: apatinib; CARB: carboplatin; LAP: lapatinib.

**Table 3:** Table summarizing the combination therapy in the past five years of resveratrol with chemotherapy in the preclinical studies in vitro (cell lines) and in vivo (rodents).

Cancer Type	Chemotherapy	Dosage	Assay Type	Molecular Effect	Study Conclusion	Ref.
Lung cancer	GEM	In vitro: 10 µM RES + 1 µM GEM. In vivo: 25 mg/kg GEM i.p. 2×/week + 1 µmol/kg RES 5×/week	HCC827 cell lines and HCC827 xenografts in nude mice	Downregulation of mRNA and protein levels of ENG, activation of ERK signaling pathway.	RES promoted tumor microvessel growth, increased blood perfusion and drug delivery into tumor that resulted in enhanced anticancer effect of GEM.	[157]
Colorectal cancer	5-FU	10 mg/kg b.w. RES p.o./day + 12.5 mg/kg b.w. 5-FU i.p. injected on days 1, 3, and 5; repeated every 4 weeks for 4 months	Methyl nitrosourea-induced colon cancer in male albino rats	Decrease of NF-κB and reduction of COX-2, induced p53 gene expression.	RES biochemically modulated and enhanced the therapeutic effects of 5-FU.	[169]
		0–200 µM RES + 10 µM 5-FU	DLD1 and HCT116 cell lines	Abolished CD44 expression, inhibition of STAT3 and Akt signaling pathways, decreased binding of STAT3 to the hTERT promoter, subsequently reduced telomerase activity.	RES enhanced the antitelomeric and pro-apoptotic potential of 5-FU in CRC, and led to re-sensitization to chemotherapy.	[170]
		5 μM RES + 1 nM 5-FU	HCT116 and HCT116R/5-FU drug-resistant cell lines	Suppressed expression of NF-κB, MMP-9 and CXCR4, induced caspase-3 cleavage, suppressed vimentin, transcription factor slug and induction of E-cadherin.	RES chemosensitizes CRC cells to 5-FU in TNF-β-induced inflammatory tumor microenvironment.	[171]
Liver cancer	CIS	12.5 μg/mL RES + 0.625 μg/mL CIS, 25 µg/mL RES + 1.25 µ/mL CIS	C3A and SMCC7721 cell lines	Reduced glutamine transporter ASCT2 expression and glutamine uptake, affected expression of cytochrome c, caspase-9 and activated caspase-3.	Synergistic effects and enhanced CIS toxicity in human hepatoma cell lines.	[158]
	SOR	In vitro: 80 μM RES + 2.5, 5, 10 μM SOR. In vivo: RES (20 mg/kg, i.p.) + SOR (25 mg/kg, p.o.) 2×/week for 3 weeks.	HepG2, Huh7 HCC cell lines and BALB/c nude mice	Accumulation of cells in S phase and decrease of G0/G1 phase, decreased levels of CDK2 and CDC25A and increased level of cyclin A, increased levels of cleaved caspase-3, caspase-8, and caspase-9 proteins, decreased expression of PKA, p-AMPK, and eEF2K.	Synergistic effects in vitro and in vivo.	[159]
		40, 80 μM RES + 0.25–10 μM SOR	Hep3b and HepG2 cell lines	-	RES potentiated the lethality of SOR.	[104]
Gastric cancer	CIS	20 μM RES + 1 μg/mL CIS	AGS cell line	Upregulation of Bax and the cleaved form of PARP, downregulation of Bcl-2, increased PERK, p-eIF2α and CHOP protein levels. Activation of PERK/eIF2α/ATF4/CHOP signaling pathway, induction of G2/M cell cycle arrest.	Synergistically inhibited cell growth of cancer cell lines.	[151]
Breast Cancer	CIS	12.5, 25, 50 μM RES + 4 μM CIS	MDA-MB-231cell lines and female BALB/c mice MDA-MB-231 xenografts	The expressions of P-AKT, P-PI3K, Smad2, Smad3, P-JNK, and P-ERK induced by TGF-β1 were reversed after RES and CIS co-treatment.	Synergistic effect on the inhibition of breast cancer cell viability, migration, and invasion in vitro; enhanced anti-tumor effect and reduced side effect of CIS in vivo.	[160]
		57.5, 72 μM RES + 18.5, 23 μM CIS	MDA-MB-231 cell line	Activation of the caspase-9 and caspase-3 enzymes, higher mitochondrial membrane depolarization.	Co-treatment induced a higher rate of apoptosis.	[161]
		0–250 μM RES + 2–50 μM CIS	MCF-7, MCF-7R, T47-D and MDA-MB-231 cell lines	Enhanced antiproliferative effect, reduction of the HR initiation complex mRNA components in MCF-7 and MCF-7R cells.	Co-treatment lowered the concentrations of CIS needed for the equivalent effect compared with CIS alone.	[162]
	DOX	30 µM RES + 100 nM DOX	MCF7 cell line	-	RES potentiated long-term toxicity of DOX, probably due to the long-term increase of apoptosis and senescence in MCF-7 cells.	[163]
		50 μmol/L RES + 4 μg/mL DOX	MCF-7 and MCF-7/ADR drug-resistant cell lines	RES reversed DOX induced upregulation of vimentin and N-cadherin and β-catenin, upregulated SIRT1 expression, reversed EMT and inhibited cell migration in MCF7/ADR cells.	RES reversed DOX-resistance in MCF-7/ADR cells.	[164]
		100, 200, 300 μM RES + 2 mg/mL DOX	MCF-7/ADR drug resistant cell line	Activation of caspase-8 and caspase-9, inhibition of proliferation and decreased cell viability, miRNA miR-122-5p upregulation and miR-542-3p downregulation, the expression levels of targeted proteins of these miRNAs significantly reduced.	RES chemo-sensitizes drug resistant cancer cell lines.	[65]
		In vitro: 10 mg/L RES + 1 mg/L DOX In vivo: 3 mg/kg DOX i.p. every week and 50 mg/kg RES p.o. for 4 weeks	MCF-7, MCF-7/DOX drug resistant cell lines and nude mice xenograft model	In vitro: PI3K and cleaved caspase-3 upregulation, reduced ratios p-Akt/Akt and p-mTOR/mTOR in MCF-7/DOX cells. In vivo: significant increase in the expression of PI3K and cleaved caspase-3, reduced p70 S6K and Ki67 expression.	In vitro, RES reversed DOX resistance, inhibited DOX-resistant breast cancer cell propagation and metastasis and facilitated cell apoptosis. In vivo, RES and DOX synergistically reduced the tumor volume.	[165]
		20 mg/kg/day RES with 2.5 mg/kg DOX in six injections for 2 weeks	MCF-7 cell line and xenografts in mice	99mTc-MIBI uptake in MCF-7 cells was significantly reduced due to higher apoptosis in tumor cells.	The combination of RES and DOX enhanced the antitumor effect and reduced DOX cardiotoxicity and hepatotoxicity.	[166]
	PTX	1 μM RES + 1, 10, 100 nM PTX	MCF-7, T47D (ERα+) and MDA-MB 231 (ERα−) cell lines	-	RES enhanced cell sensitivity to PTX and lowered the doses of PTX.	[167]

RES: resveratrol; GEM: gemcitabine; 5-FU: 5-fluorouracil; CIS: cisplatin; SOR: sorafenib; DOX: doxorubicin; PTX: paclitaxel.

**Table 4:** Table summarizing the combination therapy in the past five years of epigallocatechin gallate with chemotherapy in the preclinical studies in vitro (cell lines) and in vivo (rodents).

Cancer Type	Chemotherapy	Dosage	Assay Type	Molecular Effect	Study Conclusion	Ref.
Lung cancer	CIS	EGCG (1.5 mg/mouse/day IP) for 5 days and CIS (2 or 4 mg/kg IP) on day 5; EGCG (1.5 mg/mouse/day) and single-dose CIS (2 mg/mouse) on day 0 or 5	A549 cell xenograft bearing BALB/c nude mice	Increased CIS concentration in tumor tissue and tumor growth delay due to EGCG induced vascular normalization.	EGCG synergistically potentiated CIS antitumor efficacy especially when CIS was applied during the vascular normalization window.	[175]
		0–25 µM EGCG + 2.5 µM CIS; 15 µM EGCG + IC_90_ CIS	H460 cell line	Inhibition of ERCC1/XPF activity and the repair of CIS induced interstrand crosslinks.	EGCG enhanced sensitivity to CIS in a lung cancer cell line.	[176]
	DOX	0.5 μM EGCG + 0–100 μM DOX	Nonresponsive A549 cell line	Decreased drug efflux, MDR signaling and invasiveness. Increased drug internalization, cell cycle arrest, stress induced damage and cell death.	EGCG reversed the compromised functionality of DOX in a nonresponsive A549 cell line and improved its oxidative damage-mediated antitumor effect by modulating redox signaling.	[177]
	DOX or 5-FU	0.3 μM EGCG + 10, 20 µM 5-FU for HCT15 cells; 36 μM EGCG + 0.25, 0.5 µM DOX for A549 cells	HCT15 colon and A549 lung cancer cell lines	Increased growth inhibitory effect of 5-FU and DOX, as well as their effect on apoptosis, but not on cell cycle. EGCG sensitized 5-FU and DOX to further suppress ERK phosphorylation.	EGCG combination with DOX or 5-FU reduced cancer cell growth in different cancer cell lines.	[181]
	ETO	0.05–500 µM EGCG + 0–100 μM ETO	Nonresponsive A549 cell line	Downregulation of MRP-1 and increased intracellular uptake and retention of ETO. Suppression of MMP-2 and MMP-9 activity. EGCG helped maintain an optimum level of Nrf2 which contributed to overcoming ETO resistance.	EGCG sensitized lung adenocarcinoma cells towards ETO chemotherapy by inducing G2/M arrest and suppressing the multidrug resistance.	[178]
	GEF or OSM	10–150 µM EGCG + 1, 2.5, 5 µM GEF for PC9-GR1, PC9-GR3 and PC9-GR4 cells; 10–150 µM EGCG + 0.5, 1, 2 µM OSM for PC9-GR3 cells	PC9/GEF drug resistant cell lines: PC9-GR1, PC9-GR3 and PC9-GR4	The results shed light on the possible involvement of FASN/EGFR/STAT3 pathways.	EGCG combination with either GEF or OSM resulted in mostly additive effects.	[179]
	GEF	In vitro: 34 μM EGCG + 1.87 μM GEF; 40 µM EGCG + 10 µM GEF. In vivo: 200 mg/kg/day EGCG and 10 mg/kg/day GEF, p.o.	A549/GEF drug resistant cell line and A549 cell xenograft bearing BALB/c nude mice	Inhibition of GEF induced autophagy and ERK phosphorylation, as well as LC3-II/I and ATG5 expression, while the expression of p62 increased.	Synergistic inhibition of GEF resistant NSCLC cell proliferation and tumor growth suppression in a xenograft mouse model.	[180]
Colorectal cancer	DOX or 5-FU	0.3 μM EGCG + 10, 20 µM 5-FU for HCT15 cells; 36 μM EGCG + 0.25, 0.5 µM DOX for A549 cells	HCT15 colon and A549 lung cancer cell lines	Increased growth inhibitory effect of 5-FU and DOX, as well as their effect on apoptosis, but not on cell cycle. EGCG sensitized 5-FU and DOX to further suppress ERK phosphorylation.	EGCG combination with DOX or 5-FU reduced cancer cell growth in different cancer cell lines.	[181]
	DOX	100, 109, 117 µM EGCG + 3.63, 3.08, 3.07 µM DOX	Caco-2 cell line	EGCG decreased the dose of DOX needed to reach cytotoxicity by mediating P-gp activity.	Non-toxic EGCG concentrations combined with DOX resulted in antagonism or slight additivity in Caco-2 cell line.	[182]
	5-FU	100 µM EGCG + 0.1–100 µM 5-FU	HT-29 cell line	Significant decrease in cell proliferation.	Cotreatment enhanced the sensitivity of HT-29 cells to 5-FU by 12-fold.	[183]
	5-FU or IRI	0.2, 1, 6, 12 µM EGCG + 1, 10 µM 5-FU for KM12 cells; 0.2, 1, 6, 12 µM EGCG + 1, 10 µM 5-FU for WiDr cells; 2, 20 µM EGCG + 2, 40 µM IRI for SW837 cells	KM12, WiDr and SW837 cell lines	Synergy can only be observed in some cell lines and the underlying mechanism is possibly a combination of several mechanisms, not just a simple induction of MET.	The combination of EGCG and 5-FU resulted in synergy for WiDr cell line, while no synergy was observed for KM12 cell line. The combination of EGCG and IRI did not result in synergy for SW837 cell line.	[184]
	5-FU	In vitro: 50 µM EGCG + 1, 5, 10, 15, 20, 30 µM 5-FU. In vivo: intratumoral injection of 25 mg/kg EGCG + 20 mg/kg 5-FU for 14 successive days	HCT-116, DLD1 cell lines and DLD1 tumor bearing BALB/c nude mice	Significant enhancement of cancer cell apoptosis and DNA damage. Inhibition of GRP78 expression and increased NF-κB and miR-155-5p levels, followed by a decrease in MDR1 expression and promotion of 5-FU accumulation in cancer cells. Activation of caspase-3 and PARP, inhibition of Bcl-2 expression and increased level of Bad.	EGCG enhanced the sensitivity of colon cancer cells to 5-FU.	[185]
	IRI	In vitro: 20, 50 μM EGCG + 0.5 μM IRI. In vivo: 5 mg/kg EGCG 1 time per day, i.p. + 4 mg/kg IRI 2 times per week, i.p.	RKO, HCT116 cell lines and HCT116 tumor bearing BALB/c nude mice	Increased intracellular GRP78 expression, decreased mitochondrial membrane potential, as well as intracellular ROS and induced cell apoptosis.	EGCG increased the sensitivity of colorectal cancer cells to IRI.	[186]
		2, 5, 10, 20, 50, 100, 200 μM EGCG + 0.5 μM IRI for RKO cells. 5, 10, 20, 50 μM EGCG + 0.5 μM IRI for HCT116 cells	RKO and HCT116 cell lines	Enhanced inhibitory effect on tumor cells, induced cell apoptosis and prevention of tumor cell migration and invasion. Inhibition of topoisomerase I caused cell cycle arrest in S or G2 phase.	EGCG and IRI combination resulted in enhanced DNA damage in human colorectal cancer cells and synergistic antitumor effects.	[187]
Liver cancer	SOR	100 mg/kg EGCG + 10 mg/kg SOR	Diethyl nitrosamine induced hepatocellular carcinoma in Wistar albino rats	Histopathological observations revealed a satisfying decline in tissue degeneration and hyperchromatism. Significantly lower alpha-fetoprotein and liver enzyme levels were detected, as well as a greater antioxidant capacity.	EGCG and SOR combination had a comparable effect with standard dose SOR. The combination resulted in enhanced chemoprotection and is considered effective against hepatocellular carcinoma.	[188]
Gastric cancer	CIS	25 μg/mL EGCG + 5 μg/mL CIS	BGC-823 cell lines	Significant nuclear shrinkage and reduction in proliferation rate, cloning efficiency and cell migration. Cycle arrest in G1 phase, increased apoptosis and up-regulation of p19^Arf^, p53 and p21^Cip1^ gene and protein expression.	EGCG enhanced CIS antitumor effect against gastric cancer cells.	[189]
Breast cancer	ATO and/or irradiation	10–100 µM EGCG + 2 Gy radiation; 10–100 µM EGCG and 4 µM ATO. 10–100 µM EGCG, 4 µM ATO and 2 Gy radiation.	MCF-7 cell lines	Bax upregulation and Bcl-2 downregulation.	Combination of EGCG and ATO with or without radiation showed synergistic effects in breast cancer treatment visible in the rise of cell death.	[190]
	CLF	10 µM EGCG + 640 nM CLF for MCF-7 cells; 10 µM EGCG + 50 nM CLF for MDA-MB-231 cells	MCF-7 and MDA-MB-231 cell lines	Enhanced inhibitory effect of CLF on RARB promoter methylation and consequential induction of RARB expression. An increase in PTEN and CDKN1A transcript levels was also observed.	EGCG and CLF combination synergistically inhibited cell growth and induced apoptosis. The combination exerted a promising anticancer effect.	[191]
	SAHA	5 μM EGCG + 3 μM SAHA	ERα (+) MCF-7, ERα (−) MDA-MB-157, MDA-MB-231 and HCC1806 cell lines	Decreased expression of cIAP2 and increased expression of caspase 7. Changes in histone modifications indicate an involvement of epigenetic mechanisms in cIAP2 expression modulation. Increased apoptosis and a reduction of TNBC cell migration.	EGCG and SAHA combination successfully induced breast cancer cell apoptosis and reduced their migratory ability.	[192]
		5 μM EGCG + 3 μM SAHA	ERα (+) MCF-7, ERα (−) MDA-MB-157, MDA-MB-231 and HCC1806 cell lines	Decreased density of cancer cells and changes in expression of ERα, oncogenic miR-221/222, p27 and PTEN. Changes in histone acetylation indicate an involvement of epigenetic mechanisms in tumor suppressor expression modulation. Increased E-cadherin and decreased N-cadherin expression levels. DNMT activity and the migratory capacity of TNBC cells were reduced.	EGCG and SAHA combination successfully limited the growth, proliferation and migration of breast cancer cells.	[193]

EGCG: epigallocatechin gallate; CIS: cisplatin; DOX: doxorubicin; 5-FU: 5-fluorouracil; ETO: etoposide; GEF: gefitinib; OSM: osimertinib; IRI: irinotecan; SOR: sorafenib; ATO: arsenic trioxide; CLF: clofarabine; SAHA: suberoylanilide hydroxamic acid.

## Data Availability

Not applicable.
